# Experimental colitis in young Tg2576 mice accelerates the onset of an Alzheimer’s-like clinical phenotype

**DOI:** 10.1186/s13195-024-01471-2

**Published:** 2024-05-21

**Authors:** Luca Lorenzini, Lorenzo Zanella, Michele Sannia, Vito Antonio Baldassarro, Marzia Moretti, Maura Cescatti, Corinne Quadalti, Simone Baldi, Gianluca Bartolucci, Leandro Di Gloria, Matteo Ramazzotti, Paolo Clavenzani, Anna Costanzini, Roberto De Giorgio, Amedeo Amedei, Laura Calzà, Luciana Giardino

**Affiliations:** 1 Department of Veterinary Medical Sciences (DIMEVET), University of Bologna, Bologna, Italy; 2 IRET Foundation, Bologna, Ozzano Emilia Italy; 3 Department of Pharmacy and Biotechnology (FaBiT), University of Bologna, Via Tolara di Sopra 41/E, Bologna, 40064 Ozzano Emilia Italy; 4 Department of Experimental and Clinical Medicine, University of Florence, Florence, Italy; 5 Department of Neuroscience, Psychology, Drug Research and Child Health NEUROFARBA, University of Florence, Florence, Italy; 6 Department of Biomedical, Experimental and Clinical Sciences “Mario Serio”, University of Florence, Florence, Italy; 7 Department of Translational Medicine, University of Ferrara, Ferrara, Italy

**Keywords:** Astrocytes, Cytokines/chemokines, Gut microbiota/microbiome, Neuroinflammation, Preclinical Alzheimer’s disease, Systemic inflammation, Tg2576 mice

## Abstract

Systemic inflammation and neuroinflammation affect the natural course of the sporadic form of Alzheimer’s disease (AD), as supported by epidemiological and preclinical data, and several epidemiological studies indicate a higher prevalence of AD in patients with inflammatory bowel disease. In this study, we explored whether colitis induced by dextran sulfate sodium (DSS) in young, presymptomatic/preplaque mice worsens and/or anticipates age-dependent cognitive impairment in Tg2576, a widely used mouse model of AD. We demonstrated that DSS colitis induced in young Tg2576 mice anticipates the onset age of learning and memory deficit in the Morris water maze test. To explore potential mechanisms behind the acceleration of cognitive decline in Tg2576 mice by DSS colitis, we focused on gut microbiota, systemic inflammation and neuroinflammation markers. We observed a Firmicutes/Bacteroidetes ratio change in Tg2576 DSS animals comparable to that of elderly Tg2576 mice, suggesting accelerated microbiota aging in Tg2576 DSS mice, a change not observed in C57BL6 DSS mice. We also observed substantial differences between Tg2576 and WT mice in several inflammation and neuroinflammation-related parameters as early as 3 months of age, well before plaque deposition, a picture which evolved rapidly (between 3 and 5.5 months of age) in contrast to Tg2576 and WT littermates not treated with DSS. In detail, following induction of DSS colitis, WT and Tg2576 mice exhibited contrasting features in the expression level of inflammation-evoked astrocyte-associated genes in the hippocampus. No changes in microglial features occurred in the hippocampus between the experimental groups, whereas a reduced glial fibrillary acidic protein immunoreactivity was observed in Tg2576 vs. WT mice. This finding may reflect an atrophic, “loss-of-function” profile, further exacerbated by DSS where a decreased of GFAP mRNA expression level was detected. In conclusion, we suggest that as-yet unidentified peripheral mediators evoked by DSS colitis and involving the gut-brain axis emphasize an astrocyte “loss-of-function” profile present in young Tg2576 mice, leading to impaired synaptic morphological and functional integrity as a very early sign of AD.

**Supplementary Information:**

The online version contains supplementary material available at 10.1186/s13195-024-01471-2.

## Introduction

Alzheimer’s disease (AD) is the most frequent form of dementia, affecting over 55 million people worldwide. Recognized as a public health priority by the WHO, the pathological hallmarks of the disease include extracellular deposition of amyloid peptides into the amyloid plaques, and neural degeneration characterized by intracellular fibrillary aggregates (tangles) [1]. Two age-of-onset subtypes have been recognized: late-onset form (LOAD), and early-onset form (EOAD) prior to 65 years of age, which accounts for 5–10% of patients. Within the EOAD subtype, single-gene disorders related to several mutations of genes encoding for the amyloid precursor protein and related enzymes presenilin 1 and 2 (*APP*, *PSEN1* and *PSEN2*) account for 1% of AD cases. The recognition of mutations in amyloid-related genes has driven the theory known as the “amyloid cascade hypothesis” which has dominated the field over the last 40 years and has guided pharmacological development [2].

Most individuals, however, develop what is referred to as “sporadic” AD, characterized by a complex etiology in which multiple susceptibility genes, environmental and lifestyle factors play major roles in AD pathology, clinical onset, and disease progression [3, 4]. The pathophysiological changes leading to cognitive symptoms begin many years before the clinical manifestations of the disease, and the spectrum of AD spans from clinically asymptomatic individuals to severely impaired patients [5], an “AD continuum paradigm” which drives preclinical research involving animal models and biomarker discovery in preclinical and clinical settings. The AD continuum implies that the window for AD risk factors and the potential preventive/therapeutic options is much wider than that established by clinical AD diagnosis and includes the presymptomatic disease stage. In this framework, the Lancet Commission on Dementia Prevention, Intervention, and Care has identified 12 potentially preventable risk factors over the lifespan which may account for 40% of sporadic AD cases [6].

A large body of evidence indicates a relationship between systemic inflammation, neuroinflammation and AD [7], based on the assumption that a dysregulated immune response is a cardinal feature of AD [8]. Epidemiological data suggest a role of both acute and chronic systemic inflammation in the progression of cognitive decline in AD [9, 10]: chronic inflammatory conditions, autoimmune diseases, chronic infections (including periodontal disease), poor lifestyle factors such as chronic stress, poor sleep, sedentary lifestyle, and gut microbiota/microbiome (GM) changes (dysbiosis) all contribute to AD, while acute inflammatory conditions, surgery (orthopedic, cardiac anesthesia), acute infection (pneumonia, Covid-19, etc.) and conditions leading to tissue damage may also be implicated. Using conventional pro-inflammatory tools such as LPS and cytokines, different AD mouse models have shown an association between peripheral inflammation, amyloid pathology and microglia/astroglia status [11].

These results suggest that microglia activation and astrogliosis, both of which are activated by inflammatory and immune stimuli, are not merely epiphenomena accompanying plaque deposition and tau accumulation, instead acting as drivers of AD pathology well before the appearance of pathological AD features and clinical manifestations. Damage-associated molecular patterns (DAMPs) have been associated with central nervous system-resident immune cells (namely microglia), distinguishing the microglia phenotype in neurodegenerative diseases (such as AD) from neuroinflammatory diseases (such as multiple sclerosis) and challenging the M1/M2 paradigm [8]. New insights are challenging previously held beliefs about the role of astrocytes in AD, and the astrocyte-derived plasma glial fibrillary acidic protein (GFAP) has been proposed as an early marker of the disease, even in cognitively normal individuals with a normal amyloid-β status [12, 13].

Several epidemiological studies have suggested a higher occurrence of AD in subjects with inflammatory bowel disease (IBD), an umbrella term which includes ulcerative colitis and Crohn’s disease [14, 15]. According to a Taiwanese National Health Insurance Research Database study, the overall dementia incidence among IBD patients was significantly higher compared to controls (5.5% vs. 1.4%, respectively), and IBD patients were diagnosed with dementia 7 years earlier than those without IBD (76 vs. 83 years, respectively) [16]. Similar results were obtained in another study based on electronic health records from 26 major US health care systems, where IBD was identified as an independent risk factor for AD development, and IBD-AD patients were younger than AD patients without IBD [17]. The consolidated gut microbiota-brain axis theory also links diseases involving substantial gut GM dysregulation (as occurs in IBD) to peripheral and CNS changes, inflammatory immune activation and AD, and these data point to a major role of GM dysbiosis in cognitive CNS dysfunction when taken together [14]. While bacteria or their components, such as LPS, can permeate an impaired blood brain barrier, the main role in the bidirectional communication between the brain and the gut is played by microbial-derived metabolites, such as short chain fatty acids (SCFAs) [18].

To explore the impact of diseases characterized by systemic inflammation and gut dysbiosis on age-related cognitive decline in murine AD models, we explored whether colitis induced by dextran sulfate sodium (DSS) in young, presymptomatic/preplaque mice worsens and/or anticipates age-dependent cognitive impairment in Tg2576 mice, a widely used model of AD. We induced acute colitis in 3-month-old mice, considering that cognitive defects normally appear in this mouse strain between 6 and 10 months of age, depending on plaque deposition (which occurs between 8 and 10 months of age) and the cognitive domain explored [19]. To investigate the possible mechanisms underlying Tg2576 cognitive performance, we focused on systemic inflammation and GM dysbiosis as potential triggers/mediators of microglia/astrocyte-mediated neuroinflammation.

## Materials and methods

### Animals

We used transgenic Tg2576 mice carrying a transgene coding for the 695 amino acid isoform of human APP derived from a large Swedish family with early-onset AD (B6; SJL-Tg(APPSWE)2576Kha) and non-transgenic littermates (001349-W, WT in the text), purchased from Taconic Europe (Lille Skensved, Denmark) [20]. Animals were housed in a common animal facility at 22 ± 2 ℃, 55% humidity and a 12-h light/dark cycle, with unrestricted access to food and drinking.

All animal protocols described herein were carried out according to European Community Council Directive 2010/63/EU and Italian legislation (Legislative Decree 26/2014) and in compliance with the ARRIVE (Animal Research Reporting of In Vivo Experiments) guidelines and the NIH Guide for the Care and Use of Laboratory Animals. The project has been reviewed by the Animal Welfare Body of the IRET Foundation, and approved by the Italian Ministry of Health (authorization no. 742/2020-PR of 28/07/2020).

### Experimental design

**Fig. 1:**
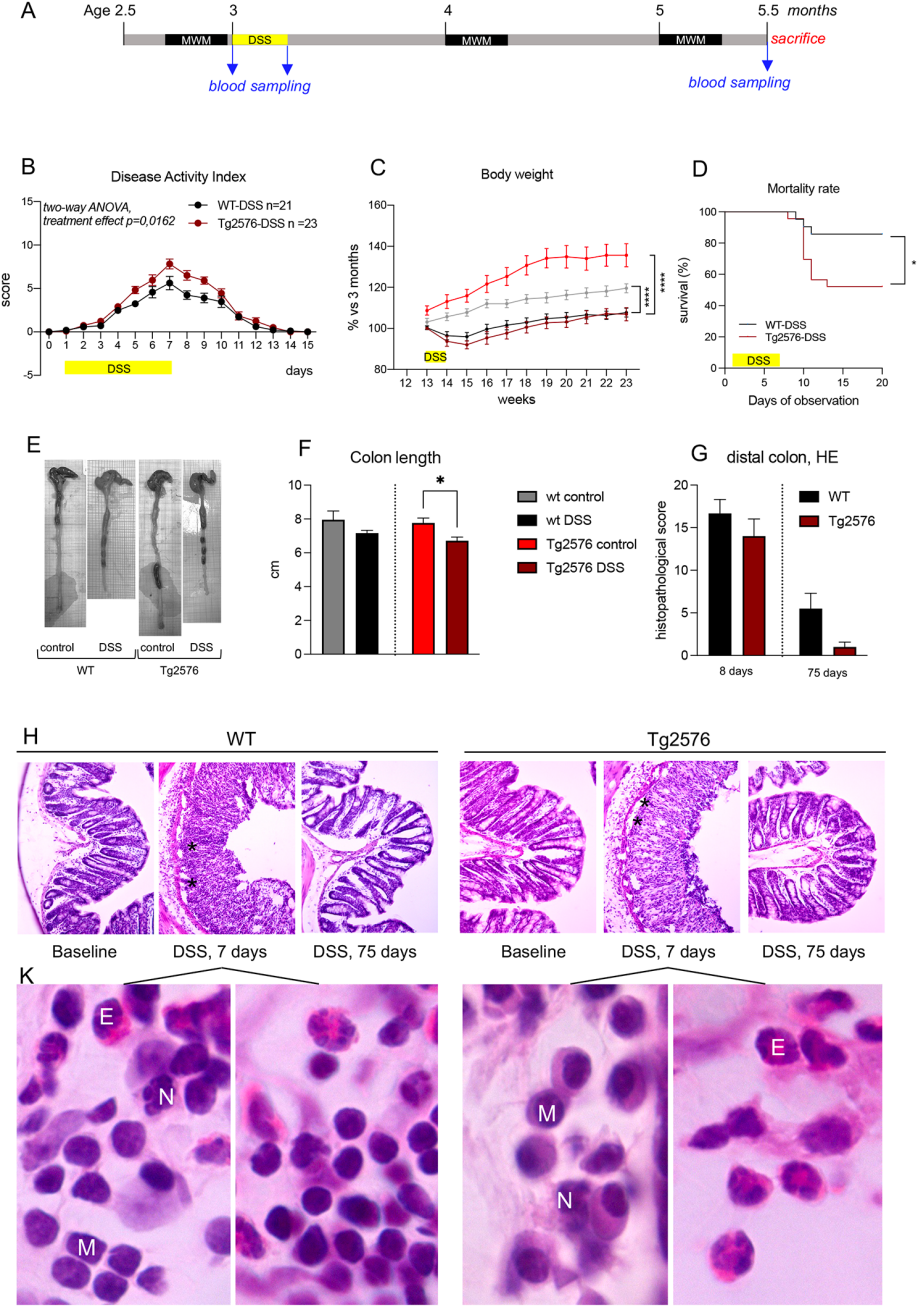
Experimental design and DSS colitis monitoring in WT and Tg2576 mice. (**A**). Experimental design of the study. Timeline of DSS treatment, blood samplings, behavioral tests, and sacrifice are shown according to the age of the mice. (**B**). Disease Activity Index (DAI); (**C**). Body weight; (**D**). Survival curve of DSS and vehicle (CTRL) mice in WT and Tg2576 strains. (**E**). Representative photos of colon at sacrifice in the experimental groups; (**F**). Colon length at sacrifice (*N* = 8–11); (**G**). Histopathology score of distal colon at sacrifice in the experimental groups (*N* = 4). (**H**). Representative micrographs of H&E sections of the colon of WT and Tg2576 mice at different time points, corresponding to baseline, clinical colitis (7 days) and sacrifice. (**K**). Representative, higher power micrographs of H&E sections of the colon of WT and Tg2576 mice clinical colitis (7 days). Data are expressed as mean ± SEM. Statistical analysis: B,C, two-way ANOVA, * *P* < 0.05; D, Gehan-Breslow-Wilcoxon test, *p* = 0.0150; Log-rank (Mantel-Cox) test,  *p* = 0.0142; F, G. Student’s t-test, * *p* < 0.05

The experimental design is shown in Fig. 1A. Since female mice are partially protected against chemically-induced colitis, only male mice were used in the study [21]. Four experimental groups were included: vehicle-treated Tg2576 mice (Tg2576 control); DSS-treated Tg2576 mice (Tg2576 DSS); vehicle-treated non-transgenic littermates (WT control), and DSS-treated non-transgenic littermates (WT DSS), all purchased from Taconic Europe. The mice performed a baseline learning and memory test using the Morris water maze at 2.5 months of age before DSS administration, then at 4 and 5 months of age, and sacrificed immediately after the final test at 5.5 months. Blood sampling was performed at baseline, during colitis and at sacrifice, and brain tissues (hippocampus and cerebral cortex) and gut collected for morphological and molecular analysis. Four additional cohorts (WT vehicle and DSS-treated; Tg2576 vehicle and DSS-treated) were used for the cytofluorimetric analysis of microglial cells and qRT-PCR neuroinflammation array in the hippocampus during the acute phase of DSS-induced colitis.

The number of animals included in each experiment is shown in the figures and/or figure legends. Possible exclusion criteria are given in each results section.

### DSS colitis induction and monitoring

Acute colitis was induced by oral administration of 1.5% DSS (36-50kD, TdB Consultancy, Sweden, MW 40,000). DSS was added to the drinking water for seven consecutive days (days 1–7), then the mice were returned to tap water [22, 23]. The control group received tap water only. All animals were observed daily and evaluated from day 1 to day 14 to calculate the disease activity index (DAI), as described in Table 1. Body weight was measured weekly over the course of the experiment.

**Table 1:** Disease Activity Index (DAI). Each parameter (body weight, stool consistency, bleeding) was scored from 0–4, giving a final score of between 0 and 12

Score	Body weight loss	Stool consistency	Bleeding
0	None	Normal	None
1	1–5%	50% normal, 50% very soft	None
2	6–10%	25% normal, 75% very soft	Presence of blood in some stools
3	11–20%	100% loose/very soft	Blood in 100% of stools
4	> 20%	Diarrhea	Gross bleeding + blood in stools

### Morris water maze

The Morris water maze is the most widely used spatial learning and memory test in AD mice models. Tests were carried out using a tank (diameter 125 cm, height 45 cm) filled with water made opaque by the addition of starch, and containing a transparent platform (10 cm diameter). The light intensity of the room was set at 130 ± 20 lx before each test session and the water temperature was 20 ± 2 °C. The tank was divided in four equal quadrants by the video tracking software ANY-Maze (ANY-Maze software v1, Stoelting Co., Wood Dale, USA), and cues positioned around the pool and on the walls of the room (visual cues).

The protocol used in this study assessed spatial learning (starting the test from different, random locations around the perimeter of the tank - acquisition phase) and reference memory (retention). Mice were brought into the test room 30 min in advance. On the first day, mice were gently placed for 30 s on the platform positioned 1 cm above the water level and made visible by flags (habituation). The subsequent acquisition phase was carried out with the platform submerged in a fixed position, and consisted of three trials per day (with a 1-hour interval between trials) for six consecutive days in 3-month-old mice, then for days in 4- and 5-month-old mice. Mice were placed in the pool facing the wall of the tank, and the latency to reach the platform area (escape latency, acquisition) was recorded. Mice unable to reach the platform within 60 s were gently accompanied on to it for habituation reinforcement. A probe trial was performed 24 h after the final acquisition day (retention), and the latency to reach the platform was recorded.

### Plasma cytokine levels

Cytokine levels were determined in plasma using a commercial kit based on Luminex xMAP technology and the Luminex 200 platform. The Mouse Cytokine/Chemokine Magnetic Bead Panel (MCYTOMAG-70 K, Merck Millipore) was used to quantify tumor necrosis factor α (TNF-α) and interleukin (IL)-6, IL-10 and IL-17. Following plasma separation, all samples were stored at < -60 °C until use. Thawed plasma samples were then centrifuged at 4000xg for 10 min at 4 °C prior to the assay and analyzed according to the manufacturer’s indications.

Briefly, undiluted samples were incubated overnight at 4 °C with antibody-immobilized beads, after which the beads were removed, and two incubations with the detection antibody and streptavidin-phycoerythrin were performed. Finally, fluorescence intensities were read on Luminex 200, and cytokine levels (expressed in pg/ml) determined by interpolation on standard 5-parameter logistic curves using xPonent Software.

### Colon histology

Histology was performed on day 0 (prior to DSS administration), day 8 (following 7d of DSS intake, corresponding to the clinical peak of inflammatory disease) and at sacrifice following the long-term experiment (corresponding to 5.5-month-old mice). On day of sacrifice, mice were deeply anesthetized with isoflurane (3–4%) in 2% O_2_ and perfused with normal saline. The colon was excised, rinsed with fresh phosphate buffered saline, measured for total length, and then fixed for 24 h in 4% paraformaldehyde and 14% picric acid in 0.2 M Sorensen buffer (pH 6.9). Colon samples were washed in 5% sucrose in 0.2 M Sorensen buffer (pH 7.4), frozen in N_2_, and coronal sections cut to 14 μm thickness using a cryostat (CM1950, Leica Biosystems) and processed with hematoxylin/eosin staining (H&E). Images were captured using a Nikon Microphot-FXA equipped with a Nikon DXM1200F CCD camera (Nikon). The histological score of colonic inflammation was blindly evaluated according to Ramakers et al. (2007) by assessing: (1) percentage of involved area; (2) number of follicles; (3) edema; (4) erosion/ulceration; (5) crypt loss; (6) infiltration of polymorphonuclear cells, and (7) infiltration of mononuclear cells. The percentage of area involved, erosion/ulceration and crypt loss were scored on a scale ranging from 0 to 4 (0 - normal; 1 - less than 10%; 2–10–25%; 3–25-50%, and 4 - >50%). Follicle aggregates were counted and scored as follows: 0 - zero to one follicles; 1 - two to three follicles; 2 - four to five follicles, and 3 - six follicles or more. The severity of the other parameters was scored on a scale from 0 to 3 as follows: 0 – absent; 1 – weak; 2 - moderate, and 3 - severe. The total score of all parameters therefore ranged from 0 to 24.

### Immunofluorescence staining and evaluation

On day of sacrifice, mice were deeply anesthetized with isoflurane (3–4%) in 2% O_2_ and perfused with normal saline. The brains were quickly dissected and immersed in 4% paraformaldehyde and 14% picric acid in 0.2 M Sorensen buffer overnight. The tissues were then washed at least five times in 0.2 M Sorensen buffer containing 5% sucrose, and frozen using liquid N_2_. Coronal cryostat sections (Leica CM1950 Biosystems Walldorf, Germany) of the brain (Bregma − 1.70 mm) with a thickness of 14 μm were collected and processed for indirect immunofluorescence using the following primary antibody: anti-GFAP (Rabbit, 1:500, Dako, cat Z0334. Santa Clara, CA); anti-Iba1 (Rabbit, 1:500, Wako Chemicals, Neuss, Germany; cat. 019-19741); 6E10 anti-Aβ1–16 monoclonal antibody (Covance, Princeton, New Jersey, USA; cat. SIG-39,320, lot. D11AF00145) for APP/Aβ (this antibody reacts with APP, sAPPβ precursor and the processed forms of Aβ) at a 1:1000 dilution. Secondary antibodies conjugated with Rhodamine Red ™-X (RRX) (1:200, Jackson Immuno Research, Cambridgeshire, UK) and Cy2 (1:200, Jackson Immuno Research, Cambridgeshire, UK) were used.

Micrographs were obtained using the Nikon Eclipse N*i* microscope fitted with the Nikon DS-Qi2 camera. Immunoreactivity analyses were performed using NIS-Elements AR software (v4.30.02, Nikon, Tokyo, Japan). Two regions of interest were measured in the CA1/2 field of the hippocampus in each mouse. The binary area fraction was calculated for each antibody, and the mean value used for the statistical analysis.

### Cytofluorometry

Briefly, the animals were deeply anesthetized with isoflurane (3–4%) in 2% O_2_, perfused with Dulbecco’s modified PBS (DPBS), and the hippocampus isolated macroscopically. To dissociate the tissue, the Adult Brain Dissociation Kit (Miltenyi Biotec, Hilden, Germany) was used, including the Red Blood Cell Removal Solution (Miltenyi Biotec) and the Debris Removal Solution (Miltenyi Biotec). Tissues were dissociated in the GentleMACS Octo Dissociator (Miltenyi Biotec).

The obtained cell suspension was marked with the live cell staining kit (Miltenyi Biotec), a mix of fluorescent molecule-conjugated antibodies designed to detect the entire microglial population (Anti-CD11b-VioGreen, Cat. 130-113-811, Miltenyi Biotec) and subfamilies (Anti-CCL2-APC, Cat. 130-107-536; Anti-CD253-VioBright 515, Cat. 130-117-567; Anti-CD88-PerCP-Vio700, Cat. 130-106-127; Anti-P2 × 7R-PE, Cat. 130-114-329; Anti-CD61-PE-Vio770, Cat. 130-122-151; Anti-CD195-APC-Vio770, Cat. 130-120-168; Miltenyi Biotec) via a direct immunofluorescence reaction. Immunolabeled cell count analysis was performed using MACSQuant Analyzers and FlowLogic software (Miltenyi Biotec). Live cells were first extracted based on size and live cell staining, then the entire microglial/macrophage population quantified using the CD11b marker. From the CD11-positive cell group, cells double positive for the other markers were quantified to detect the different subpopulations.

### Gene expression analysis

Gene expression analysis was performed in the hippocampus of WT control, WT DSS, Tg2576 control and Tg2576 DSS animals (*n* = 5–6 per group) at sacrifice. 3-month-old WT and Tg2576 mice were also included in this experiment.

The total RNA was extracted from the homogenized tissues using the RNeasy Plus Universal Mini Kit (Qiagen, Hilden, Germany) following the manufacturer’s instructions and quantified by Nanodrop 2000 spectrophotometer (Thermo Fisher Scientific). For cDNA synthesis, the RT^2^ first strand kit (Qiagen) was used, with 1 µg (1000 ng) of pooled RNAs from each experimental group. Eighty-four genes involved in the inflammation process were analyzed using the commercial RT^2^ PCR array PAMM-011ZD (Qiagen), which also included five common housekeeping genes and qPCR quality controls (see supplementary Table 1 for the complete gene list). Following the manufacturer’s protocol, the real-time PCR was performed using the RT^2^ SYBR Green qPCR Mastermix (Qiagen). The whole data sets passed all quality controls, taking inter-plate reproducibility, reverse transcription reaction and genomic DNA contamination into account.

The isolated RNAs were also used for the qPCR gene expression analysis of disease-associated microglia genes (*Trem2*, *Nfe2l2*, *P2ry12*, *Tmem119*, *Cx3cr1*, *Nlrp3*, *Apoe*) in the hippocampus of WT control, WT DSS, Tg2576 control and Tg2576 DSS animals (*n* = 5 per group). Reverse transcription was performed using the iScript™ gDNA Clear cDNA Synthesis Kit (Bio-Rad Laboratories, Segrate, MI, Italy), starting with 1 µg of RNA and following the manufacturer’s instructions.

A no-reverse transcription sample was added, using the iScript Supermix No-RT Control instead of the reverse transcription enzyme, and processed for gene expression analysis in parallel with the other samples to check for genomic DNA contaminations. The qPCR reaction was performed using the CFX96 machine (Bio-Rad) and the SsoAdvanced™ Universal SYBR®Green Supermix (Bio-Rad), starting with 10 ng of cDNA. Relative quantification of mRNA was obtained using the comparative cycle threshold (Cq) method. Cq values were collected for each sample and normalized on the housekeeping gene glyceraldehyde 3-phosphate dehydrogenase (*GAPDH*). Specific primers were used to analyze the expression of the target genes (Table 2), with a tested efficiency approximate to 2. The formula 2^-(ΔΔCq) was used as a semi-quantification method, initially normalizing the Cq of the target gene with the housekeeping gene expression (ΔCq) and subsequently with a control group (ΔΔCq), as specified in the figure legend for each graph. The relative expression is then shown as a fold change relative to the control group.

**Table 2:** Sequences of the forward and reverse primers used to analyze gene expression via qPCR

Primer	Forward (5’-3’)	Reverse (3’-5’)
*Trem2*	AAAGTACTGGTGGAGGTGCTG	TCTTGATTCCTGGAGGTGCTG
*Nfe2l2*	CTGAACTCCTGGACGGGACTA	CGGTGGGTCTCCGTAAATGG
*P2ry12*	AAAATGCCTGCTGCTTGAAT	TGAAGAAATTCCAACAAAACGA
*Tmem119*	GTGTCTAACAGGCCCCAGAA	AGCCACGTGGTATCAAGGAG
*Cx3cr1*	CCGGTCTCATTTGCAGGCTTA	TGGGAATTGAACTTGGGACCTC
*Nlrp3*	GTGGAGATCCTAGGTTTCTCTG	GAGGACCTCATTCTCTTGGATC
*ApoE*	CAGTGGCCCAGGAGAATCAA	GAGACTCAGAATGTGCTCGGA
*GFAP*	AGTGGTATCGGTCCAAGTTTGC	TGGCGGCGATAGTCATTAGC
*Gapdh*	GGCAAGTTCAATGGCACAGTCAAG	CATACTCAGCACCAGCATCAC

### Fecal SCFA evaluation by GC-MS analysis

Qualitative and quantitative evaluation of fecal SCFAs (acetic, propionic, butyric, isobutyric, 2-methylbutyric, isovaleric and valeric acids) was performed using an Agilent gas chromatography-mass spectrometry (GC-MS) system consisting of a single quadrupole mass spectrometer (model 5971), gas chromatograph (model 5890) and autosampler (model 7673), using our previously described method [24].

Briefly, fecal pellets were thawed just before analysis and added to a 1.5 mL centrifuge tube with 0.25 mM sodium bicarbonate solution (1:1 w/v). The obtained suspensions were then sonicated for five minutes, centrifuged at 5000 rpm for 10 min and the supernatants collected. The SCFAs were then extracted as follows: a 100 µL aliquot of sample solution (corresponding to 0.1 mg of stool sample) was added to 50 µL of internal standards mixture, 1 mL of tert-butyl methyl ether and 50 µL of HCl 6 M + 0.5 M NaCl solution in a 1.5 mL centrifuge tube. Each tube was then shaken in a vortex apparatus for two minutes and centrifuged at 10,000 rpm for five minutes. The solvent layer was then transferred to an autosampler vial and processed three times.

### Fecal microbiota characterization

Genomic DNA was extracted using the DNeasy PowerSoil Pro Kit (Qiagen, Hilden, Germany) from frozen (-80 °C) fecal pellets, according to the manufacturer’s instructions. Briefly, 5–7 fecal pellets for each sample were added to a bead-beating tube and homogenized with TissueLyser LT (Qiagen, Hilden, Germany) for five minutes at 50 Hz. The DNA was then captured on a silica membrane in a spin column format, washed and eluted. The quality and quantity of the extracted DNA were assessed using both NanoDrop ND-1000 (Thermo Fisher Scientific, Waltham, USA) and Qubit Fluorometer (Thermo Fisher Scientific, Waltham, USA) systems, then frozen at -20 °C. Total DNA samples were then sent to IGA Technology Services (Udine, Italy), where amplicons of the variable V3–V4 region of the bacterial 16 S rRNA gene (341 F: CCTACGGGNGGCWGCAG; 805R: GACTACNVGGGTWTCTAATCC) were sequenced in paired-end mode (2 × 300 cycles) on the Illumina MiSeq platform, according to the Illumina 16 S Metagenomic Sequencing Library Preparation protocol.

Demultiplexed sequence reads were processed using QIIME2 2022.8. The sequencing primers and reads without primers were removed using the Cutadapt tool v3.4, while DADA2 was used for filtering, merging and removing chimeras from paired-end reads after trimming low-quality nucleotides from both forward and reverse reads. Amplicon sequence variants (ASVs) were then generated, and the taxonomic assignments performed using the Scikit-learn multinomial ® Bayes classifier re-trained on the SILVA database (release 138) V3-V4 hypervariable region.

### Statistical analysis

The statistical analyses on bacterial communities were performed in R 4.1.0 using the phyloseq 1.38.0, vegan 2.6-2, DESeq2 1.32.0 and other packages satisfying their dependencies. The ggplot2 3.3.6 package was used to plot data and results. Each genus with a maximum relative abundance of less than 0.01% was removed to avoid probable contaminants and sequencing errors [25, 26]. A saturation analysis on ASV was performed on every sample using the rarecurve function (step 100 reads), and further processed to highlight saturated samples (arbitrarily defined as saturated samples with a final slope in the rarefaction curve with an increment in ASV number per reads < 1e-5). The observed richness and Shannon indices were used to estimate the bacterial alpha diversity in each sample using the estimate_richness function on phyloseq. Pielou’s evenness index was calculated using the formula E = ®og(R), where S is the Shannon diversity index and R is the observed ASV richness in the sample. Differences in alpha diversity indices and Firmicutes/Bacteroidetes ratio were inspected using the Mann-Whitney test. PCoAs were performed using the Hellinger distance on Hellinger transformed genera abundances. PERMANOVA and Betadisper were used to test the statistical significance of the beta diversity distances and dispersions. At different taxonomic ranks, the differential analysis of the abundances was computed with DESeq2 on raw count data. To limit noisy results, differentially abundant taxa with a DESeq2 baseMean value < 50 were discarded from the displayed results, irrespective of their statistical significance. Further details on the bacterial community analyses are available at:

https://github.com/LeandroD94/Papers/tree/main/2024_AD_gut_Mice_C57BL6_Tg2576_DSS.

The statistical analyses of all other graphs and data were generated using GraphPad Prism v8.2.1 (GraphPad Software, San Diego, CA, USA), and all data are expressed as mean ± SEM. Student’s t-test was used to compare the means between two groups, while ANOVA and post-hoc tests were used to compare the means between three or more groups. Results were considered significant when the probability of their occurrence due to chance alone was less than 5% (*p* < 0.05).

## Results

### DSS-induced colitis

According to the conventional protocol, 1.5% DSS was administered in drinking water for 7 days to both genotypes. DSS is a sweet tester, that is preferred by the strains included in this study [27]. To guarantee the same DSS intake in WT and Tg2576 mice, we then measured water consumption in Tg2576 and WT mice, and no differences were observed between genotype (supplementary material Table 2). This DSS concentration induced mild colonic inflammation. Colitis-related symptomatology was evaluated via the DAI score (Fig. 1B) and body weight measurements (Fig. 1C). DAI scores progressively increased in all DSS-treated animals during DSS administration in both WT DSS and Tg2576 DSS groups, and these animals showed a similar pattern of recovery on DSS withdrawal until disappearance of symptoms at day 14. Tg2576 mice displayed slightly more severe symptoms than WT (two-way ANOVA, *p* = 0.0162).

Body weight was lower in Tg2576 compared to WT animals, as expected (Supplementary Fig. 1). Results related to DSS administration are shown as a percentage variation compared to baseline, and showed a 10% reduction during DSS administration, with no differences between WT and Tg2576 mice (Fig. 1C). Two weeks after DSS withdrawal, the growth curves resumed the typical trend for these mouse strains, but remained much lower than those of vehicle-treated mice (two-way ANOVA, ***p < 0.001, ****p < 0.0001). A significantly higher percentage of DSS-induced mortality was observed in Tg2576 (50%) compared to WT mice (10%) (Fig. 1D) (Gehan-Breslow-Wilcoxon test, *p* = 0.0261; Log-rank (Mantel-Cox) test, *p* = 0.0222).

The effect of DSS administration was also evaluated based on colon length at the end of the experiment, with results showing a shorter colon in Tg2576 DSS-treated compared to vehicle-treated mice (Fig. 1E and F). Histological analysis of the distal colon was performed on day 8 after DSS withdrawal, and at the end of the experiment (75 days after DSS discontinuation), evaluating crypt loss and infiltration of polymorphonuclear and mononuclear cells. Results showed acute and extensive DSS-induced epithelial erosion and ulceration, with mono- and polynuclear white cells infiltration into the lamina propria and submucosa as detected in H&E stating, which returned to normal following DSS withdrawal (Fig. 1G–K).

### DSS colitis worsens age-dependent cognitive decline in Tg2576 mice

To determine whether DSS colitis induced at a young age anticipates the onset or worsens the severity of learning and memory defects, mice were tested in the Morris water maze (MWM) at 3, 4 and 5 months of age (Fig. 1A). Studies report that learning and memory defects in the MWM test do not normally appear prior to 5.5-6 months of age [19].

**Fig. 2:**
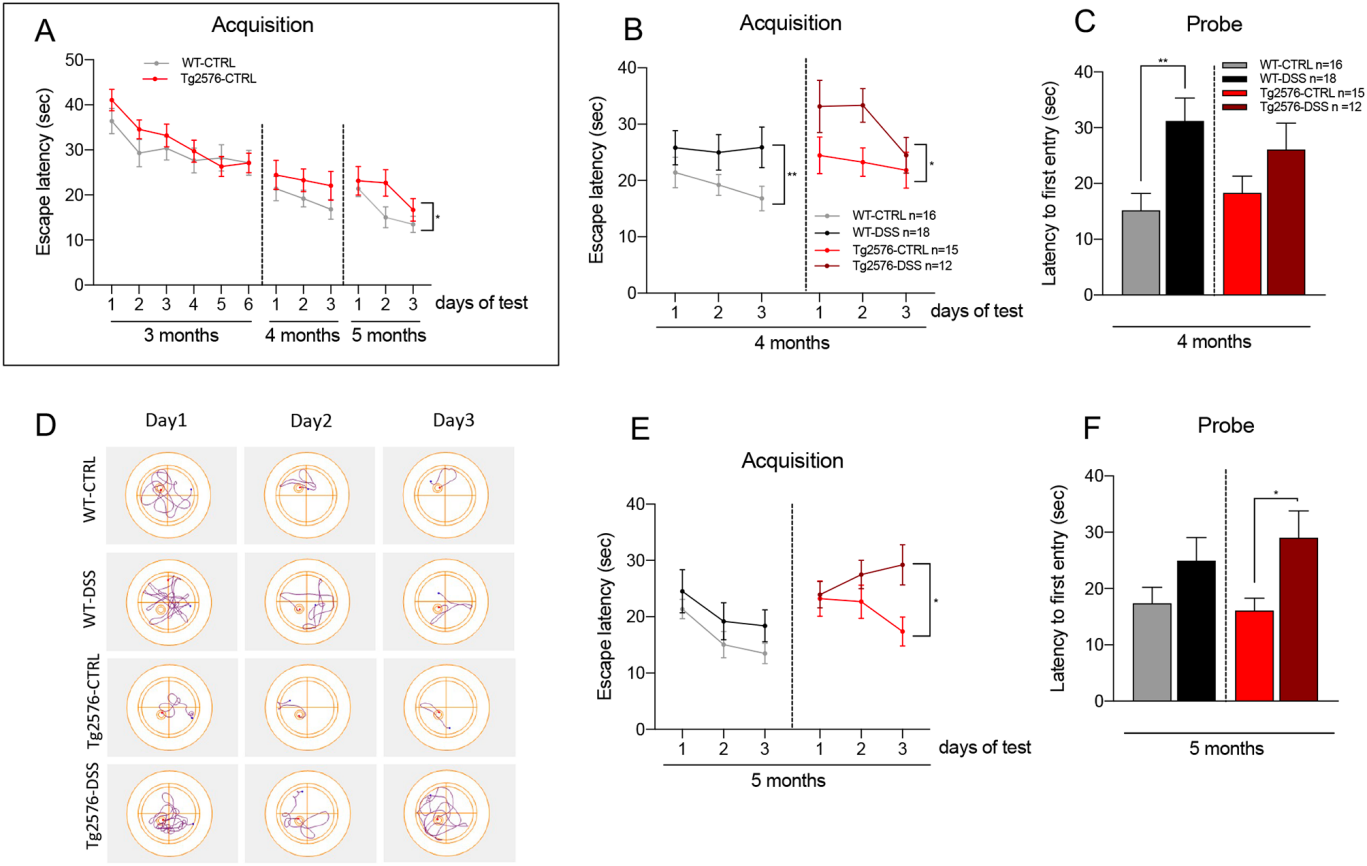
Effect of DSS treatment on MWM performance at different age-points. (**A**). Acquisition phase at 3, 4 and 5 months on control groups, expressed as latency to reach the submerged platform. (**B**). Acquisition phase at 4 months in WT, WT DSS, Tg2576 and Tg2576 DSS groups, expressed as latency to reach the submerged platform. (**C**). Probe trial in 4-month-old mice, on all groups, expressed as latency to the first entry into the platform area. (**D**). Representative track plots of acquisition trial of mice at 5 months of age in different groups. (**E**). Acquisition phase at 5 months in WT control, WT DSS, Tg2576 control and Tg2576 DSS groups, expressed as latency to reach the submerged platform. (**F**). Probe trial in 5-month-old mice, on all groups, expressed as latency to the first entry into the platform area. Data are expressed as mean ± SEM. The number of mice included in each group is shown in the legend. Statistical analysis: two-way ANOVA (**B**, **E**) and Student’s t-test (**C**, **F**), (* *p* < 0.05; ** *p* < 0.01)

Results are shown in Fig. 2, where graph A shows the characterization model based on genotype. No differences in spatial learning between the two strains were observed in vehicle-treated animals at month 3 or 4, while a slight deterioration in performance by Tg2576 compared to WT mice was observed at month 5 (two-way ANOVA, *p* = 0.0344). Both DSS-treated WT and Tg2576 mice showed a substantial impairment in the acquisition phase compared to their controls at month 4 (two-way ANOVA, *p* = 0.0076 and *p* = 0.0103 for WT and Tg2576, respectively) (Fig. 2B), a result confirmed for WT DSS mice in the probe trials (unpaired t-test, *p* = 0.0043) (Fig. 2C).

Spatial performance indices were completely restored at month 5 in C57BL6 DSS mice, and no differences compared to WT vehicle were observed either in the acquisition or probe trials, while Tg2576 DSS performance was severely impaired in both acquisition (Fig. 2D, two-way ANOVA *p* = 0.0172) and probe trials (Fig. 2E, unpaired t-test, *p* = 0.0168) (Fig. 2D).

Distance traveled and mean speed were also measured to ensure that DSS colitis did not impair the motor component of MWM performance (**Supplementary**, Fig. 2A and B). While Tg2576 mice traveled a slightly longer distance at a higher speed, as already described in this mouse strain [28, 29], no differences were observed between DSS-treated or control in either genotype. Interestingly, thigmotaxis time, considered an anxiety or fear indicator associated with an elevated level of corticosteroid [30], increased in both DSS-treated WT and Tg2576 mice, albeit to a much lower extent in the latter (Supplementary Fig. 2C).

### Young, preplaque Tg2576 mice showed altered inflammation and neuroinflammation markers compared to WT

**Fig. 3:**
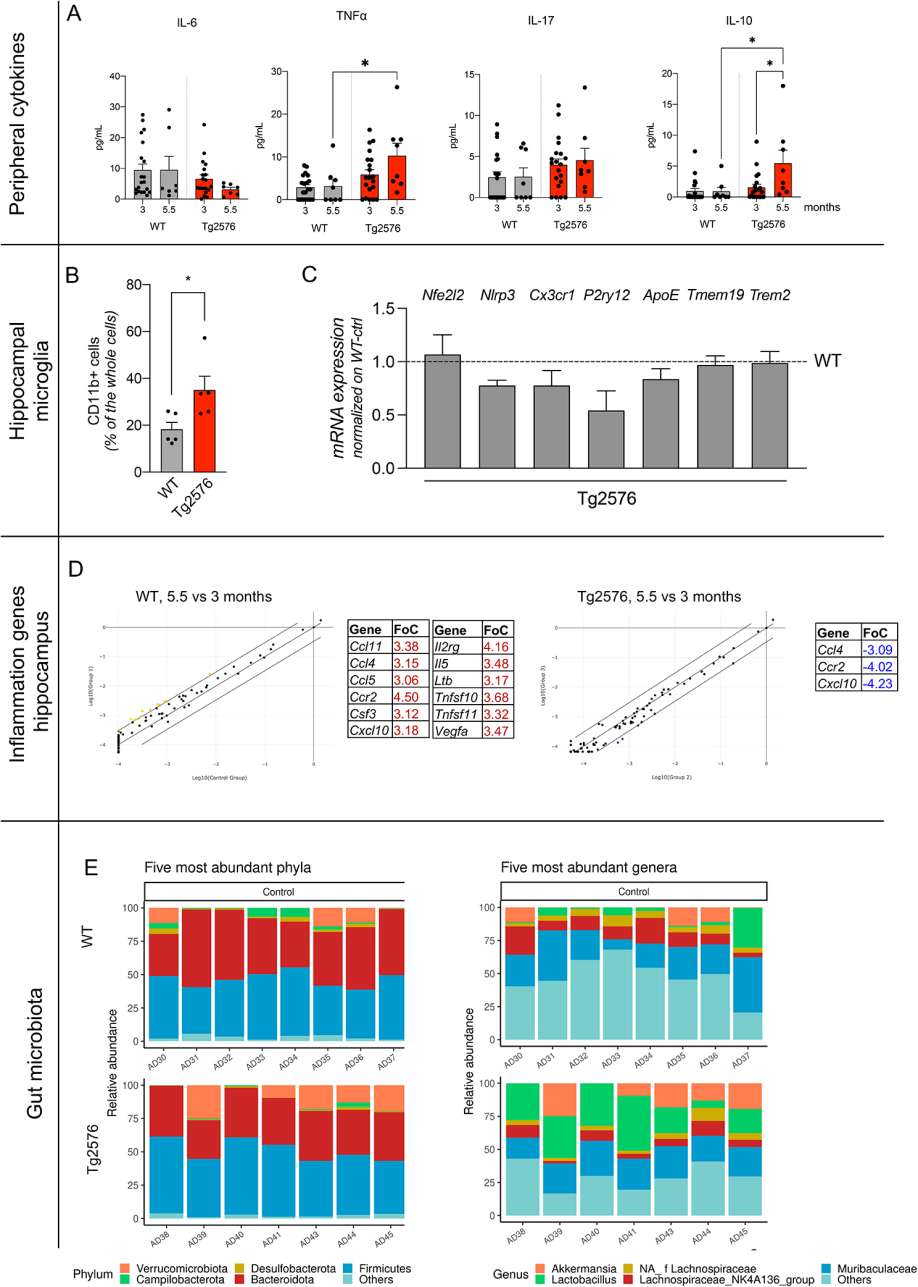
Inflammation and neuroinflammation markers in young, preplaque Tg2576 mice. (**A**). Cytokine plasma levels in 3 and 5.5-month-old WT and Tg2576, vehicle and DSS-treated mice. (**B**). Number of CD11 + microglial cells in the hippocampus in 3-month-old mice. (**C**). Expression level of disease-associated microglial genes in the hippocampus of young Tg2576 mice, expressed as a percentage variation compared to age-matching Tg2576 mice. (**D**). Scatter plots derived from 84 inputs obtained from qPCR array plates for inflammation-related genes, showing age-regulated genes (5.5 vs. 3-month-old mice) in WT and Tg2576 mice. Significance was set at a fold change of 3 (FoC). Regulated genes are listed in the table as up-regulated (red) and down-regulated (blue). (**E**). Relative abundances of the five most represented phyla and genera observed in Tg2576 and WT mice. Statistical analysis: **A**, **C**: two-way ANOVA and post-hoc Tukey’s test, * *p* < 0.05; B: Student’s t-test, * *p* < 0.05

We initially characterized the molecular and cellular biomarkers of inflammation, neuroinflammation and the gut microbiota-brain axis in Tg2576 and WT mice at the age time points included in the study. Cytokine plasma levels currently used to monitor DSS colitis (TNFα, IL6, IL10 and IL17 [23]) were dosed at 3 (baseline) and 5.5 months (Fig. 3A) to evaluate systemic inflammation. Plasma levels of the tested cytokines were the same at 3 and 5.5 months in WT mice. In 5.5-month-old Tg2576 mice, IL10 and TNFα plasma levels were higher in Tg2576 compared to the C57BL6 mice of the same age (*p* < 0.05).

We then monitored neuroinflammation in the hippocampus, a brain area deeply involved in determining MWM performance. The number of CD11b^+^-microglial/macrophage cells [31] was higher in Tg2576 mice (Fig. 3B, P < 0.05), while the mRNA expression level, analyzed in the whole tissue, of genes encoding for marker proteins for disease-associated microglia [32] did not differ between WT and Tg2576 mice (Fig. 3C).

Moreover, we quantified the percentage of microglial subpopulations using six different markers (CCL2, CCR5, CD61, CD88, P2 × 7R, CD253). All the chosen markers are associated with the activated microglia phenotype and, with the CD11b co-expression, identify specific subpopulations. In fact, all of them have been identified as upregulated in AD patients and/or AD animal models (CCL2 [33], CCR5 [34], CD61 [35], CC88 [36], P2 × 7R [37], CD253 [38]) However, in line with the subpopulation-specific gene expression, none of the analyzed double positive cells ratio resulted modified within experimental groups, due to the expression of hAPP mutated gene and/or DSS induction (Supplementary Fig. 3).

Based on the age-related changes in peripheral inflammatory cytokines, and the altered molecular signature of microglial cells already evident in the 3-month-old mice, we analyzed the changes in the expression of 84 inflammatory cytokine-related genes in the hippocampal tissue of WT and Tg2576 animals at 3 and 5.5 months. For biological averaging and variance reduction purposes, samples from each group were pooled for microarray experiments, since pooling dramatically improves accuracy for very small designs [39]. Results are presented as scatter plot showing the list of up-regulated (red) and down-regulated (blue) genes in 5.5 vs. 3-month-old C57BL6 and Tg2576 mice respectively (Fig. 3D). The significance threshold was set to a fold change of 3 (FoC). We found that 12 of the 84 investigated genes were up-regulated between 3 and 5.5 months in WT mice (*Ccl11, Ccl4, Ccl5, Ccr2, Csf3, Cxcl10, Il2rg, Il5, Ltb, Tnfsf10, Tnfsf11, Vegfa*) while none were down-regulated. In Tg2576 mice, on the other hand, only down-regulated genes characterized age-related changes (*Ccl4, Ccr2, Cxcl10*), all of which are among the up-regulated genes in WT.

### DSS-induced long-term gut microbiota alterations

The GM taxonomic analysis performed in Tg2576 and WT mice (both control and 5.5-month-old mice) showed the five most abundant phyla to be Bacteroidota, Campilobacterota, Desulfobacterota, Firmicutes and Verrucomicrobiota, while the five most abundant genera were Akkermansia, Lachnospiraceae_NK4A136, Lactobacillus and Muribaculaceae, and an unidentified genus of Lachnospiraceae family (Fig. 3E). No statistically significant differences in alpha diversity indices (observed ASV richness, Shannon index, evenness) were observed. Furthermore, although the principal coordinate analysis (PCoA) highlighted differences in bacterial communities between Tg2576 and WT mice groups (PERMANOVA, p adj.= 0.0024), no taxa were differentially abundant between the two groups (Supplementary Fig. 4 and Table 3).

**Fig. 4:**
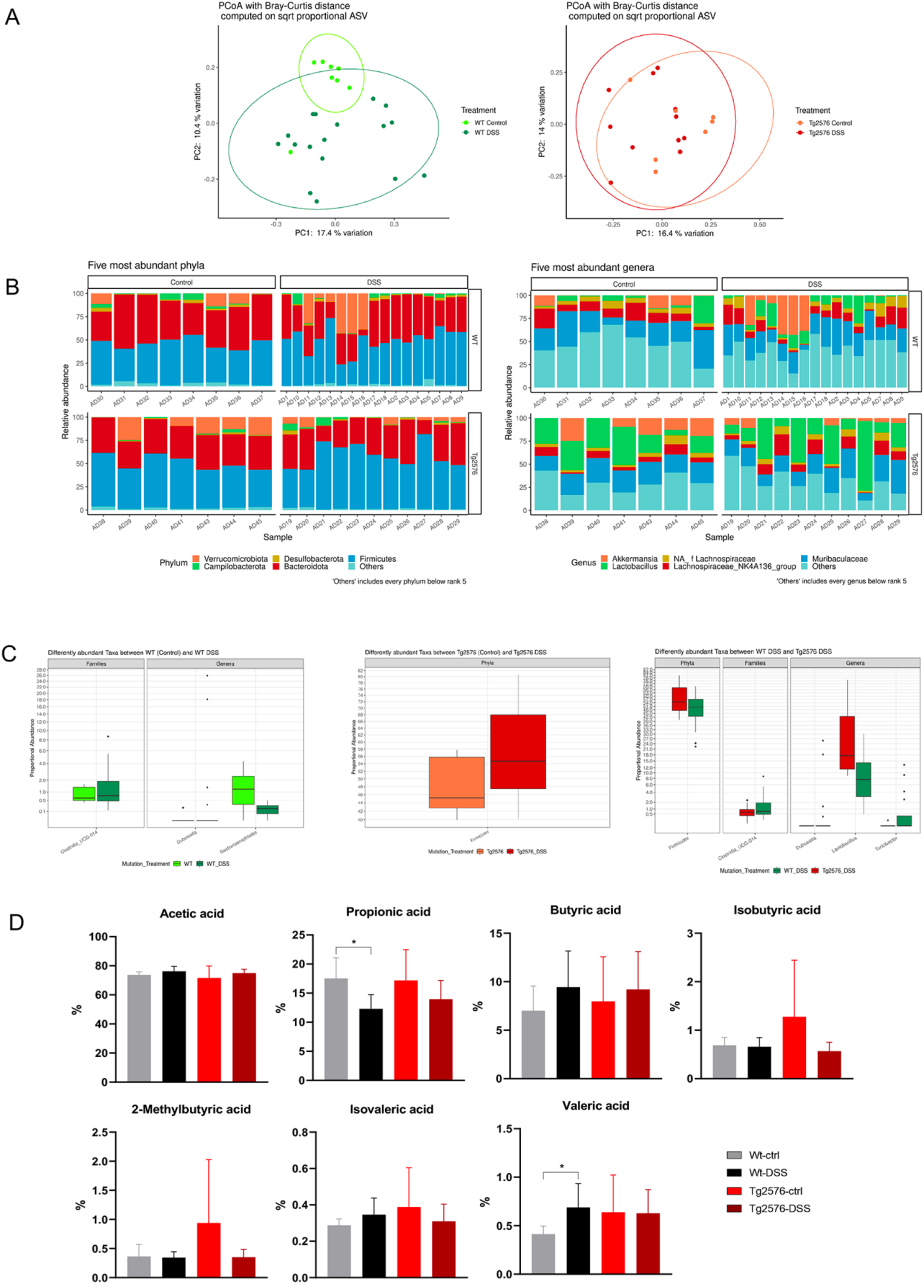
Gut dysbiosis by DSS colitis differs in Tg2576 and WT mice. (**A**). PCoAs, according to the Bray-Curtis beta diversity metric, of stool samples of WT and WT DSS mice and Tg2576 and Tg2576 DSS mice. (**B**). Relative abundances of the five most represented phyla and genera observed in WT and WT DSS mice and in Tg2576 and Tg2576 DSS mice. (**C**). Box plots showing significant differentially abundant taxa between groups. All data have a p adj.<0.05. (**D**). Bar plots showing fecal SCFAs abundances between groups. Analyses were assessed using the Kruskal-Wallis test and p adj. values less than 0.05 were considered statistically significant. * *p* < 0.05, ** *p* < 0.01, *** *p* < 0.001

Vice versa, as shown by the PCoAs, we observed that DSS induced alterations of the gut microbiota structure in WT mice only (p adj.= 0.0068), while no separation was foundbetween Tg2576 and Tg2576 DSS samples (p adj.= 0.1492) (Fig. 4A). Moreover, although no significant differences in the alpha diversity indices were observed in either group, the stacked bar plots show different DSS-associated fecal relative abundances of the top five phyla and top five genera for both WT and Tg2576 mice (Fig. 4B). In detail, compared to untreated mice, WT DSS animals showed a significant increase of members from the Clostridia_UCG-014 family (log2FC= -1,844; p adj.= 0,0178) and Dubosiella genus (log2FC= -27,6096; p adj.= 2,03e-9) but significantly reduced levels of Gastranaerophilales spp. (log2FC = 3,0871; p adj.= 0,68e-4) (Fig. 4C).

We also observed that in comparison to WT DSS animals, Tg2576 DSS mice showed significantly higher levels of bacteria from the Firmicutes phylum (log2FC = 1,1837; p adj.= 0,0128) and Lactobacillus genus (log2FC = 2,4121; p adj.= 0,0227) but decreased abundances of Clostridia_UCG-014 (log2FC= -2,0614; p adj.= 0,0259), Dubosiella spp. (log2FC= -34,2619; p adj.= 2,68e-18) and Turicibacter spp. (log2FC= -6,4412; *p* adj.= 0,0245) (Fig. 4C).

Finally, functional analysis of the GM showed lower levels of propionic acid (*p* = 0,0184) and a higher abundance of valeric acid (*p* = 0,0469) in WT DSS compared to WT mice (Fig. 4D), while Tg2576 DSS mice surprisingly showed no alterations.

### Peripheral inflammation and neuroinflammatory molecular signature in the hippocampus differ in WT and Tg2576 mice following DSS colitis resolution

Mice were sacrificed as soon as the learning and memory defect appeared in Tg2576 mice at 5.5 months of age. At this time, the DSS colitis was completely resolved, and cytokine plasma levels did not differ between control and DSS colitis groups in the respective genotypes (Supplementary Fig. 5).

**Fig. 5:**
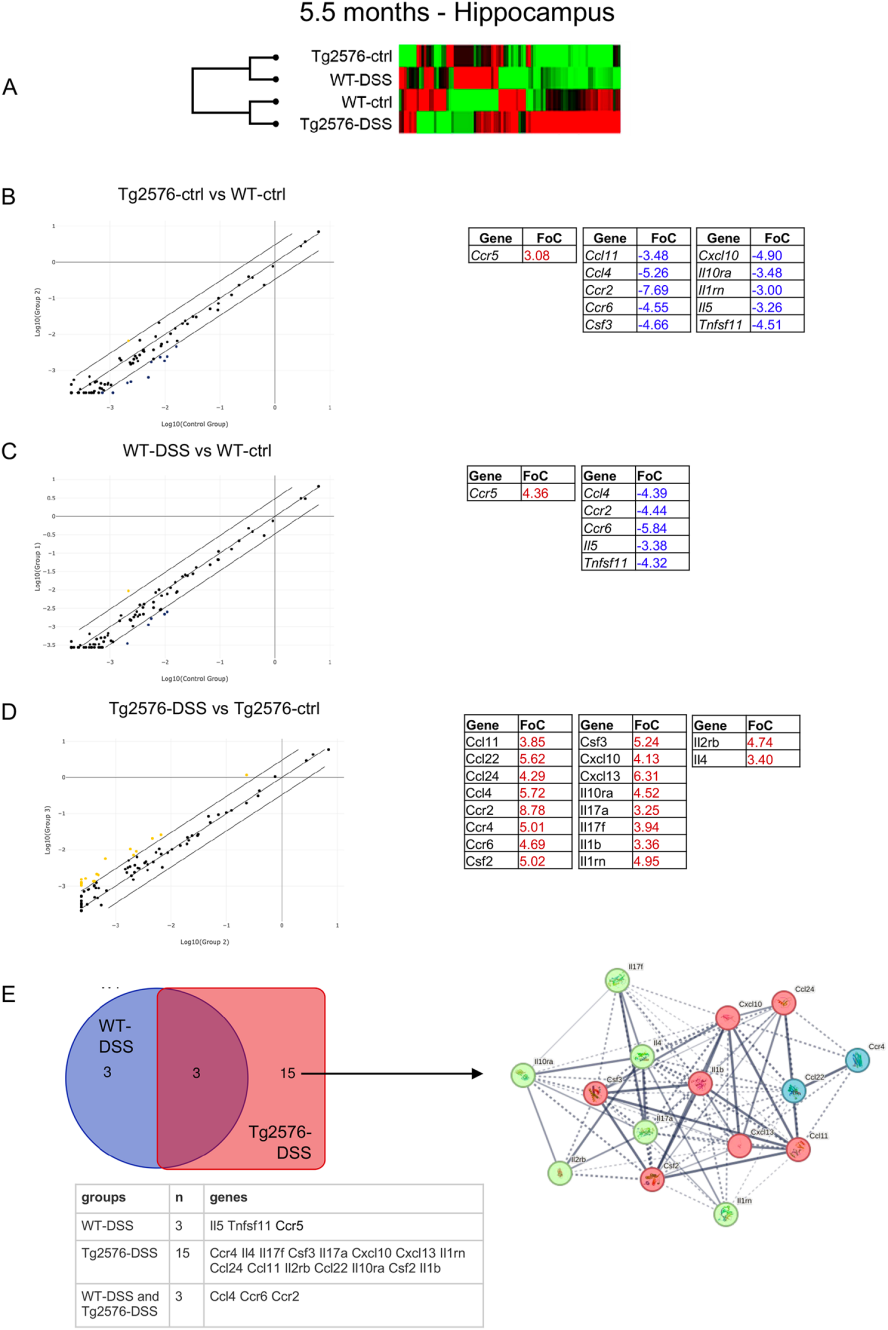
Neuroinflammation molecular signature by DSS colitis in the hippocampus of Tg2576 and WT mice. (**A**). Cluster gram analysis of the 84 inputs obtained from qPCR array plates for inflammation-related genes in the hippocampus. (**B**). Scatter plot representation of the gene expression fold change in Tg2576 control vs. WT control in 5.5 month-old mice, using a fold change of three as the significance cutoff value for gene expression variation. Regulated genes are listed in the table as up-regulated (red) and down-regulated (blue). (**C**). Scatter plot representation of the gene expression fold change in WT DSS vs. WT control mice, using a fold change of three as the significance cutoff value for gene expression variation. Regulated genes are listed in the table as up-regulated (red) and down-regulated (blue). (**D**). Scatter plot representation of the gene expression fold change in Tg2576 DSS vs. Tg2576 control mice, using a fold change of three as the significance cutoff value for gene expression variation. Regulated genes are listed in the table as up-regulated (red) and down-regulated (blue). (**E**). Venn diagram of the diversely regulated genes in WT (blue circle) and Tg2576 (red square) control mice. A fold change of two as the significance cutoff value for gene expression variation was used for this analysis. The table shows the genes listed in each interaction. The STRING interaction network of the protein encoded by genes showing a fold of change higher than two is also shown. The left net refers to genes regulated by DSS in both WT and Tg2576 mice, while the right net refers to genes regulated in Tg2576 mice only

To explore whether peripheral factors (in this case systemic inflammation and/or microbiota changes induced by DSS) evoke neuroinflammation and contribute to the worsening of cognitive status in Tg2576 mice, we analyzed the changes in the expression of 84 inflammatory cytokine-related genes in the hippocampus of WT and Tg2576 control and DSS mice a long time after DSS induction (Fig. 5). Overall results are shown as a heat map, and analysis of the expression profile of cytokine-related genes revealed that the WT control, Tg2576 DSS, Tg2576 control and WT DSS groups all form part of the same cluster (Fig. 5A).

Up-regulated (red) and down-regulated (blue) genes are shown as scatter plots (Fig. 5B, C, D). Significance threshold was set to a fold change of 3 (FoC). We initially compared control Tg2576 vs. WT at 5.5 months, showing that only the *Ccr5* gene was up-regulated, while 10 genes (*Ccl11, Ccl4, Ccr2, Ccr6, Csf3, Cxcl10, Il10ra, Il11m, Il5, Tnfsf11*) were down-regulated (Fig. 5B). WT DSS mice showed an increased expression of one gene (*Ccr5*) and a decreased expression of five genes (*Ccl4*, *Ccr2*, *Ccr6*, *Il5*, *Tnfsf11*) (Fig. 5C). Analysis of gene expression in the hippocampus of Tg2576 DSS vs. Tg2576 control mice generated the opposite result, with 18 up-regulated genes (*Ccl11, Ccl2, Ccl24, Ccl4, Ccr2, Ccr4, Ccr6, Csf2, Csf3, Cxcl10, Cxcl13, Il10ra, Il17a, Il17f, Il1b, Il1rn, Il4*) (Fig. 4D).

We also performed a functional enrichment analysis (https://string-db.org/*)* and a bioinformatic analysis to generate a Venn diagram (https://bioinformatics.psb.ugent.be/webtools/Venn/*)*, as shown in Fig. 4E, observing DSS-dysregulated genes in WT (*N* = 3, blue circle, see also associated table) and Tg2576 animals (*N* = 15, red square, see also associated table), and in both groups (*N* = 3, intersection). 2FoC genes were included in this analysis. Comparison of the STRING analysis between dysregulated genes in Tg2576 and the dysregulated genes in both WT and Tg2576 genes reveals a cluster including Il1b, Csf2, Csf3, Ccl11, Ccl24, Cxcl10, Cxcl13 (in red) which was present in Tg2576 DSS, but not in WT DSS.

**Fig. 6:**
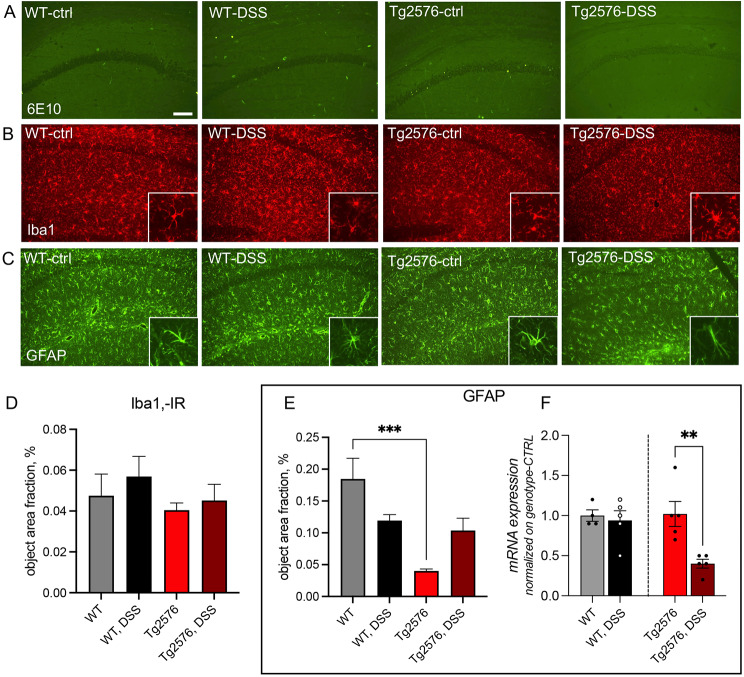
Amyloid histopathology, microglia and astroglial cells in the CA1/2 hippocampal fields of Tg2576 and WT, control, and DSS-treated mice. Amyloid plaque deposition was analyzed by 6E10-IR (**A**), microglia by Iba1 (**B**), and astrocytes by GFAP (**C**). GFAP- (**D**) and Iba1-IR (**E**) were quantified as % area fraction. *GFAP* mRNA expression level in the hippocampus was normalized vs. the WT control group (**F**). Data are expressed as mean + SEM. Statistical analysis: one-way ANOVA and post-hoc Tukey’s test, ***  *p* < 0.001 (D, E); Student’s t-test, ** *p* < 0.01 (**F**). Bar: 100 μm

Finally, we explored amyloid plaque deposition and the morphological features of microglia and astrocytes in the CA1-2 field of the hippocampus in 5.5-month-old mice, therefore a long time after DSS colitis resolution. No plaques were observed (Fig. 6A), and the microglia cell density (Fig. 6D) and morphological features (Fig. 6B) did not differ between groups. In contrast, we observed a reduction of GFAP-IR in the hippocampus of Tg2576 control compared to Tg2576 control mice (Fig. 6E). At mRNA level, there were no differences between WT and Tg2576 at baseline (C57BL6-CTRL and Tg2576-CTRL). However, at 5.5 months, the DSS colitis down-regulates GFAP gene expression specifically in Tg2576 animals (C57BL6-DSS and Tg2576-DSS, Fig. 6F).

## Discussion

Sporadic AD is a systemic, multifactorial disease, and peripheral inflammation and GM dysbiosis are thought to be factors which contribute to the natural course of the disease, acting well before the appearance of the pathological (amyloid plaques and neurofibrillary tangles) and clinical markers of the disease [3–5, 40]. Our study was designed to explore the potential of a gut inflammation model resembling human IBD (DSS colitis) induced in young Tg2576 mice to anticipate or worsen cognitive decline. DSS colitis is a widely used translational model for IBD involving systemic inflammation, GM dysbiosis, and leaky gut (increased intestinal-blood barrier permeability).

### DSS colitis anticipates the onset of learning and memory deficit in Tg2576 mice

The Tg2576 mouse is a very well characterized AD model, widely used to explore neuropathophysiology and develop pharmacological interventions. Cognitive decline in the MWM appears between 5 and 6 months in our experimental conditions, while plaque deposition occurs between 8 and 10 months [19, 29]. Most of the animal studies designed to investigate the role of LPS or cytokine-induced peripheral inflammation on amyloid pathology and related cognitive symptoms have been performed in elderly mice, when cognitive symptoms are already present [41]. To our knowledge, the very few studies using peripheral inflammation models more suitable for translation than LPS have involved a single time point, and have failed to monitor disease progression via cognitive tests. Sohrabi et al. [42] induced DSS colitis in App^*NL−G−F*^ mice, a strain which develops early cortical amyloidosis and cognitive impairment at 2 and 6 months respectively, and which showed an increased insoluble Aβ–40/42 levels and decreased microglial CD68 immunoreactivity compared to vehicle-treated mice. In the same mouse, Kaneko et al. [43] demonstrated that IBD models induced when plaques and cognitive decline were already present worsened AD plaque deposition.

Here we demonstrated that DSS colitis induced in 3-month-old Tg2576 mice anticipated the age of onset of learning and memory deficit in the MWM. In particular, the impairment observed at 4 months (close to colitis resolution) had resolved one month later in WT, while still persistent and/or more severe in Tg2576. The latter group showed severely impaired acquisition phase and probe trial results, despite body weight and locomotion parameters suggesting good overall health.

The possible correlation between systemic inflammation and neuroinflammation in the preclinical stages of AD is still elusive. To explore the possible mechanisms underlying the acceleration of cognitive decline in Tg2576 mice by DSS colitis, we focused on gut dysbiosis, systemic inflammation and neuroinflammation markers.

### Gut dysbiosis by DSS in Tg2576 and WT mice

Consistent GM alterations of the most represented phyla and genera abundances have been documented following DSS administration in mice [44]. In detail, a remarkable intestinal dysbiosis was reported in Tg2576 DSS mice, which showed a significant increase of Firmicutes when compared with both Tg2576 control and WT DSS animals. As well documented, Firmicutes (along with Bacteroidota) is one of the most dominant intestinal phyla, and the Firmicutes/Bacteroidota (F/B) ratio is used as an index of compositional GM changes.

Specifically, studies have shown Firmicutes and Bacteroidota levels to be increased and reduced in IBD patients respectively, confirming the detrimental effect of DSS on gut microbiota [45]. In accordance with our own findings, Honarpisheh and colleagues [46] found no significant difference in presymptomatic young (6-month-old) Tg2576 mice, while higher levels of Firmicutes were reported in symptomatic elderly (15-month-old) Tg2576 mice compared to 6-month-old mice and wildtype controls. We speculate that DSS colitis accelerates Tg2576 GM aging by altering the Firmicutes/Bacteroidetes ratio. Although with contrasting results [47], this ratio has been associated with cognition, including the transition from mild cognitive impairment (MCI) to AD in humans [48, 49]. In comparison to the WT DSS group, Tg2576 DSS mice also showed a significant increase in Lactobacillus, a genus belonging to the Firmicutes phylum. Considering the alterations in GM composition as AD progresses, some studies have reported a significant increase in Lactobacillus spp. between the presymptomatic and symptomatic stages of the pathogenesis in Tg2576 mice [50]. On the other hand, many Lactobacillus-based psychobiotics have demonstrated beneficial therapeutic effects in cognitive disorders, including AD, as they restore a healthy gut-brain axis communication, although different microbial strains may exert different effects on the host [51, 52].

Moreover, Tg2576 DSS mice reported significantly reduced levels of Clostridia_UCG-014, Dubosiella spp. and Turicibacter spp. In line with our results, a significantly decreased abundance of bacteria belonging to the Clostridia_UCG-014 family has been documented in relation to increased cognitive frailty index scores [53]. Notably, the abundance of both Turicibacter and Dubosiella genera was also decreased in APP^swe^/PS1^ΔE9^ mice [54].

Our functional analysis of the GM was prompted by research findings showing the ability of SCFAs to ameliorate certain AD hallmarks in mouse models [55], however our results, somewhat surprisingly, showed only reduced levels of anti-inflammatory propionic acid and a higher abundance of valeric acid in WT DSS mice compared to WT controls, while no differences were observed for Tg2576 DSS animals.

### Possible mechanisms: systemic inflammation and astrocyte dysfunction

Most of the studies investigating the role of neuroinflammation in AD cognitive decline have involved middle-aged/elderly mice, and differences in cellular and molecular inflammation-related signatures between transgenic and WT mice have been generally interpreted as a “reaction” of microglia and astrocytes to amyloid pathology [8, 32, 56–58].

Here we observed substantial differences between Tg2576 and WT mice in several inflammation and neuroinflammation-related parameters at 3 months of age, therefore well before plaque deposition, a picture which evolves rapidly up to 5.5 months of age in opposing directions in Tg2576 animals and their WT littermates. TNFa and IL10 plasma levels were higher in 5.5-month-old Tg2576 mice compared to WT, and IL10 increased in Tg2576 mice between 3 and 5.5 months. Twelve of the 84 investigated inflammation-related genes were up-regulated in the hippocampus in WT mice between 3 and 5.5 months, while none were down-regulated. On the contrary, only down-regulated genes characterized age-related changes in Tg2576 mice. Notably, the genes down-regulated in Tg2576 (*Ccl4, Ccr2, Cxcl10*) were among the up-regulated genes in WT.

Despite the higher number of CD11b-positive cells in Tg2576 compared to WT mice, none of the inflammation-related genes modified by aging in control Tg2576 mice, such as P2ry12, Csf3, Cxcr1, ApoE, Trem2, are included in microglia-associated molecular phenotypes in AD mouse models [32, 59]. Instead, most of the genes differentially expressed between 3.5 and 5 months in Tg2576 mice are preferentially associated with astrocytes, such as *Ccl4* [60] and *Ccr2* [61], a deficiency of which impairs astroglial signaling for microglial accumulation and accelerates the progression of AD-like disease [62], and *Cxcl10* [63], the dysregulation of which has been associated with cognitive defects in aging [64].

This molecular signature is consistent with the hypothesis that an astroglial dysfunction contributes to pathogenesis in sporadic AD [12, 65–67], and may indicate a functional impairment of astrocytes following DSS administration. Coherently, we observed a decreased GFAP-IR in the hippocampus of Tg2576 mice, probably reflecting an atrophic, “loss-of-function” profile [68], with DSS further reducing its mRNA expression level. GFAP down-regulation in the CNS has been associated with systemic inflammations due to infections, such as chronic infection with HIV-1 [69], varicella zoster [70], and pseudorabies [71], as well as in astrocytes derived from iPSCs obtained from SAD and FAD patients [72].

Early astrocyte dysfunction disrupts the normal synaptic microenvironment, contributing to very early synaptic dysfunction and consequent learning and memory defects [66]. We already demonstrated that at 5 months of age, when a contextual memory impairment is already present in Tg2576 mice, a long-term potentiation (LTP) impairment is registered in the hippocampus [73], associated with a lower K+-evoked [^3^H] acetylcholine release from cortical synaptosomes [74]. GM alterations such as those observed in Tg2576 DSS mice also reduce GFAP ^+^ reactive astrocytes in young APP-PS1-21 mice, both in a microglial-dependent and independent manner [75]. Astrocyte homeostasis disruption in Tg2576 mice leading to an inappropriate response to systemic risk factors (such as peripheral inflammation or gut-leaking-derived substances) may explain the cognitive decline we observed. Astrocytes are involved in the structural remodeling and functional plasticity of synapses, are engaged in the regulation of synaptic function supporting learning and memory processes [76], and may integrate environmental factors to fine-tune neural circuits in a context-specific, feedforward and adaptive fashion [77]. A breakdown in homeostatic functions has been described in gray matter astrocytes in AD brains by the transcriptomic, whose molecular profile is predominantly characterized by down-regulation of homeostatic genes [78], and the decrease in astroglial reactivity parallels the switch from MCI to AD with senile dementia [72].

Astrocyte dysfunction may be due to intracellular amyloid accumulation and/or soluble amyloid toxicity. In previous studies, we demonstrated that neonatal astrocytes derived from Tg2576 mice contain amyloid peptides [79], as detected by the 6E10 antibody [80]. Intracellular APP/Aβ40-42-amyloid peptide immunoreactivity progressively increases not only in neurons, but also in astrocytes in Tg2576 mice, reaching the highest concentration at 5 months and then decreasing as soon as extracellular plaque deposition begins [81]. This is not surprising, since the hamster prion protein promoter driving hAPP expression in Tg2576 mice is also expressed by astrocytes [82]. These results have been confirmed by other researchers, who have also excluded APP/Aβ40-42-amyloid peptide in microglia [83]. Intracellular amyloid peptide accumulation is toxic for cells, disturbing the cytoskeleton dynamic and evoking oxidative stress, leading to organelle dysfunction and metabolic defects in astrocytes [65, 84]. Soluble Aβ also evokes cell-wide astrocytic calcium dysregulation in the absence of amyloid plaques in vivo [85], another voltage-gated channel is precociously altered in Tg2576 mice astrocytes and derived cells [86].

## Conclusions

In this study, we demonstrated that peripheral systemic inflammation mimicking IBD worsened age-dependent cognitive decline when induced in young, presymptomatic Tg2576 mice. To our knowledge, this is the first study demonstrating this remarkable association.

Altered homeostatic function in astrocyte-dependent synapses in very young Tg2576 mice caused by astrocyte dysfunction probably underlies this effect. Although this study was not designed to identify possible mechanisms, we suggest that peripheral and as-yet unidentified mediators evoked by DSS colitis emphasize the astrocyte “loss-of-function” profile in Tg2576 mice. In light of the established role of the GM structure in regulating astrocyte function, the GM dysbiosis observed in Tg2576 DSS animals, which resembles the microbiota composition of elderly, cognitively impaired Tg2576 mice, may contribute to establishing a chronic astrocyte dysfunction, similar to that observed in patients with AD.

### Electronic supplementary material

Below is the link to the electronic supplementary material.

Supplementary Material 1

## Data Availability

The data presented in this study are deposited in the NCBI Gene Expression Omnibus (GEO) repository, accession number GSE253108.
