# Supplementary material for: Experimental colitis in young Tg2576 mice accelerates the onset of an Alzheimer’s-like clinical phenotype
Source: Alzheimers Res Ther. 2024 May 21;16:116. doi: 10.1186/s13195-024-01471-2 (PMC11110243; doi:10.1186/s13195-024-01471-2)
Supplement: Supplementary file 1 — Supplementary Material 1 [file 13195_2024_1471_MOESM1_ESM.docx]

| **GenBank** | **Symbol** | **Description** |
| --- | --- | --- |
| NM_007926 | *Aimp1* | Aminoacyl tRNA synthetase complex-interacting multifunctional protein 1 |
| NM_007553 | *Bmp2* | Bone morphogenetic protein 2 |
| NM_011329 | *Ccl1* | Chemokine (C-C motif) ligand 1 |
| NM_011330 | *Ccl11* | Chemokine (C-C motif) ligand 11 |
| NM_011331 | *Ccl12* | Chemokine (C-C motif) ligand 12 |
| NM_011332 | *Ccl17* | Chemokine (C-C motif) ligand 17 |
| NM_011888 | *Ccl19* | Chemokine (C-C motif) ligand 19 |
| NM_011333 | *Ccl2* | Chemokine (C-C motif) ligand 2 |
| NM_016960 | *Ccl20* | Chemokine (C-C motif) ligand 20 |
| NM_009137 | *Ccl22* | Chemokine (C-C motif) ligand 22 |
| NM_019577 | *Ccl24* | Chemokine (C-C motif) ligand 24 |
| NM_011337 | *Ccl3* | Chemokine (C-C motif) ligand 3 |
| NM_013652 | *Ccl4* | Chemokine (C-C motif) ligand 4 |
| NM_013653 | *Ccl5* | Chemokine (C-C motif) ligand 5 |
| NM_009139 | *Ccl6* | Chemokine (C-C motif) ligand 6 |
| NM_013654 | *Ccl7* | Chemokine (C-C motif) ligand 7 |
| NM_021443 | *Ccl8* | Chemokine (C-C motif) ligand 8 |
| NM_011338 | *Ccl9* | Chemokine (C-C motif) ligand 9 |
| NM_009912 | *Ccr1* | Chemokine (C-C motif) receptor 1 |
| NM_007721 | *Ccr10* | Chemokine (C-C motif) receptor 10 |
| NM_009915 | *Ccr2* | Chemokine (C-C motif) receptor 2 |
| NM_009914 | *Ccr3* | Chemokine (C-C motif) receptor 3 |
| NM_009916 | *Ccr4* | Chemokine (C-C motif) receptor 4 |
| NM_009917 | *Ccr5* | Chemokine (C-C motif) receptor 5 |
| NM_009835 | *Ccr6* | Chemokine (C-C motif) receptor 6 |
| NM_007720 | *Ccr8* | Chemokine (C-C motif) receptor 8 |
| NM_011616 | *Cd40lg* | CD40 ligand |
| NM_007778 | *Csf1* | Colony stimulating factor 1 (macrophage) |
| NM_009969 | *Csf2* | Colony stimulating factor 2 (granulocyte-macrophage) |
| NM_009971 | *Csf3* | Colony stimulating factor 3 (granulocyte) |
| NM_009142 | *Cx3cl1* | Chemokine (C-X3-C motif) ligand 1 |
| NM_008176 | *Cxcl1* | Chemokine (C-X-C motif) ligand 1 |
| NM_021274 | *Cxcl10* | Chemokine (C-X-C motif) ligand 10 |
| NM_019494 | *Cxcl11* | Chemokine (C-X-C motif) ligand 11 |
| NM_021704 | *Cxcl12* | Chemokine (C-X-C motif) ligand 12 |
| NM_018866 | *Cxcl13* | Chemokine (C-X-C motif) ligand 13 |
| NM_011339 | *Cxcl15* | Chemokine (C-X-C motif) ligand 15 |
| NM_009141 | *Cxcl5* | Chemokine (C-X-C motif) ligand 5 |
| NM_008599 | *Cxcl9* | Chemokine (C-X-C motif) ligand 9 |
| NM_009909 | *Cxcr2* | Chemokine (C-X-C motif) receptor 2 |
| NM_009910 | *Cxcr3* | Chemokine (C-X-C motif) receptor 3 |
| NM_007551 | *Cxcr5* | Chemokine (C-X-C motif) receptor 5 |
| NM_010177 | *Fasl* | Fas ligand (TNF superfamily, member 6) |
| NM_008337 | *Ifng* | Interferon gamma |
| NM_008348 | *Il10ra* | Interleukin 10 receptor, alpha |
| NM_008349 | *Il10rb* | Interleukin 10 receptor, beta |
| NM_008350 | *Il11* | Interleukin 11 |
| NM_008355 | *Il13* | Interleukin 13 |
| NM_008357 | *Il15* | Interleukin 15 |
| NM_010551 | *Il16* | Interleukin 16 |
| NM_010552 | *Il17a* | Interleukin 17A |
| NM_019508 | *Il17b* | Interleukin 17B |
| NM_145856 | *Il17f* | Interleukin 17F |
| NM_010554 | *Il1a* | Interleukin 1 alpha |
| NM_008361 | *Il1b* | Interleukin 1 beta |
| NM_008362 | *Il1r1* | Interleukin 1 receptor, type I |
| NM_031167 | *Il1rn* | Interleukin 1 receptor antagonist |
| NM_021782 | *Il21* | Interleukin 21 |
| NM_145636 | *Il27* | Interleukin 27 |
| NM_008368 | *Il2rb* | Interleukin 2 receptor, beta chain |
| NM_013563 | *Il2rg* | Interleukin 2 receptor, gamma chain |
| NM_010556 | *Il3* | Interleukin 3 |
| NM_133775 | *Il33* | Interleukin 33 |
| NM_021283 | *Il4* | Interleukin 4 |
| NM_010558 | *Il5* | Interleukin 5 |
| NM_008370 | *Il5ra* | Interleukin 5 receptor, alpha |
| NM_010559 | *Il6ra* | Interleukin 6 receptor, alpha |
| NM_010560 | *Il6st* | Interleukin 6 signal transducer |
| NM_008371 | *Il7* | Interleukin 7 |
| NM_010735 | *Lta* | Lymphotoxin A |
| NM_008518 | *Ltb* | Lymphotoxin B |
| NM_010798 | *Mif* | Macrophage migration inhibitory factor |
| NM_021524 | *Nampt* | Nicotinamide phosphoribosyltransferase |
| NM_001013365 | *Osm* | Oncostatin M |
| NM_019932 | *Pf4* | Platelet factor 4 |
| NM_009263 | *Spp1* | Secreted phosphoprotein 1 |
| NM_013693 | *Tnf* | Tumor necrosis factor |
| NM_008764 | *Tnfrsf11b* | Tumor necrosis factor receptor superfamily, member 11b (osteoprotegerin) |
| NM_009425 | *Tnfsf10* | Tumor necrosis factor (ligand) superfamily, member 10 |
| NM_011613 | *Tnfsf11* | Tumor necrosis factor (ligand) superfamily, member 11 |
| NM_023517 | *Tnfsf13* | Tumor necrosis factor (ligand) superfamily, member 13 |
| NM_033622 | *Tnfsf13b* | Tumor necrosis factor (ligand) superfamily, member 13b |
| NM_009452 | *Tnfsf4* | Tumor necrosis factor (ligand) superfamily, member 4 |
| NM_009505 | *Vegfa* | Vascular endothelial growth factor A |
| NM_007393 | *Actb* | Actin, beta |
| NM_009735 | *B2m* | Beta-2 microglobulin |
| NM_008084 | *Gapdh* | Glyceraldehyde-3-phosphate dehydrogenase |
| NM_010368 | *Gusb* | Glucuronidase, beta |
| NM_008302 | *Hsp90ab1* | Heat shock protein 90 alpha (cytosolic), class B member 1 |

**Supplementary Table 1.** List of the analyzed genes included in the PCR array plate “mouse inflammatory cytokines & receptors” (Qiagen, cat. PAMM-011Z). The table includes the GenBank code, the gene symbol, and the extended name for each of the inflammatory-related genes and the five proposed housekeeping (*Actb*, *B2m*, *Gapdh*, *Gusb*, *Hdp90ab1*).

|  |  |  |  | **BASELINE** | **64 HOURS** | **TOTAL WATER CONSUMPTION** | | | **MEAN WATER CONSUMPTION PER GENOTYPE**  **ml/die/gr b.w** | |
| --- | --- | --- | --- | --- | --- | --- | --- | --- | --- | --- |
| ID | Genotype | Body weight | Age (weeks) | Water (ml) | Water (ml) | Water intake (ml) | Total ml/h | Total ml/die/gr b.w | WT | Tg2576 |
| 784 | WT | 18,1 g | 15 | 175,9 | 158 | 17,9 | 2,46 | 3,31 | 0,34 | 0,33 |
| 787 | WT | 18,5 g | 15 | 180 | 157,1 | 22,9 | 2,45 | 2,57 |  |  |
| 786 | Tg2576 | 13,4 g | 15 | 173,7 | 158,7 | 15 | 2,47 | 3,96 |  |  |
| 836 | Tg2576 | 13,7 g | 15 | 170,3 | 159,2 | 11,1 | 2,48 | 5,37 |  |  |
| 474 | Tg2576 | 20,4 g | 35 | 198,7 | 179,9 | 18,8 | 2,81 | 3,58 |  |  |
| 475 | Tg2576 | 20,1 g | 35 | 200,3 | 185,3 | 15 | 2,89 | 4,63 |  |  |
| 429 | WT | 22,8 g | 35 | 197 | 181,2 | 15,8 | 2,83 | 4,30 |  |  |
| 430 | WT | 23,1 g | 35 | 192 | 175,4 | 16,6 | 2,74 | 3,96 |  |  |

**Supplementary Table 2.**

Water consumption over a 64 hours period was monitored in WT and Tg2576 mice housed in metabolic cages. Mice of different age and body weight were included. The water consumption was then expressed as ml/day/body weight, showing non differences between genotypes.

**Supplementary Figure 1.** Body weight at baseline in C57BL6 and WT mice.

**Supplementary Figure 2.**

Total distance travelled (A), mean speed (B), and thigmotaxis time during the probe trial in the Morris water maze.

Statistical analysis: one-way ANOVA and Tukey’s post-hoc test, *P<0.05, ** P <0.01.


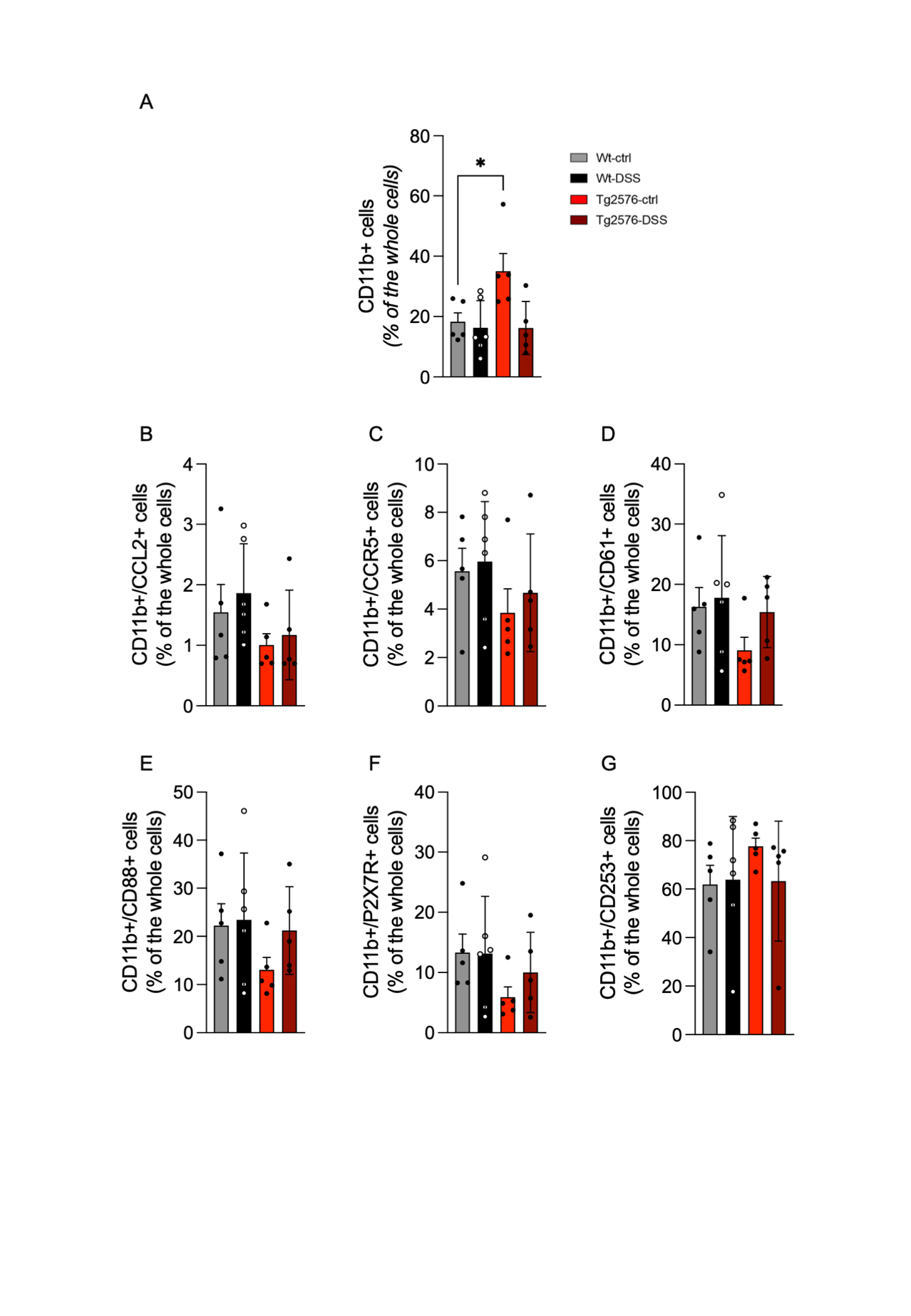


**Supplementary Figure 3**. Quantification of microglia/macrophages subpopulations, based on CD11b expression (A) and double expression of CD11b with specific microglial markers (B-G). Statistical analysis: One-way ANOVA and post-hoc Tukey’s test, * P < 0.05.


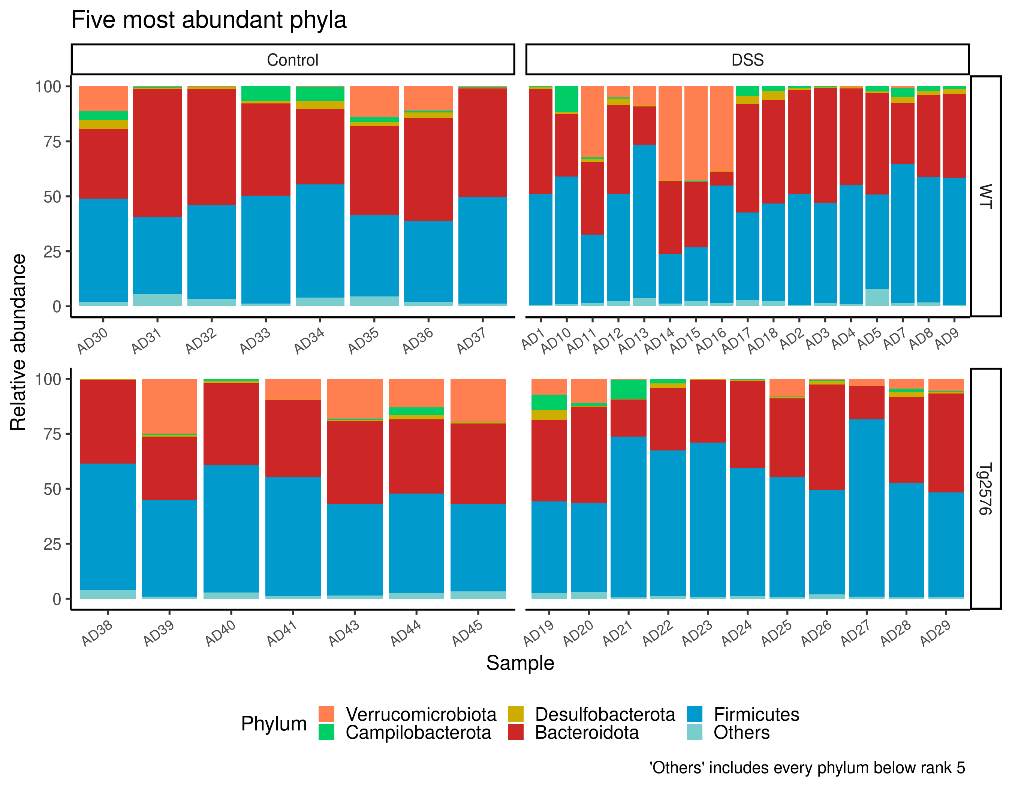

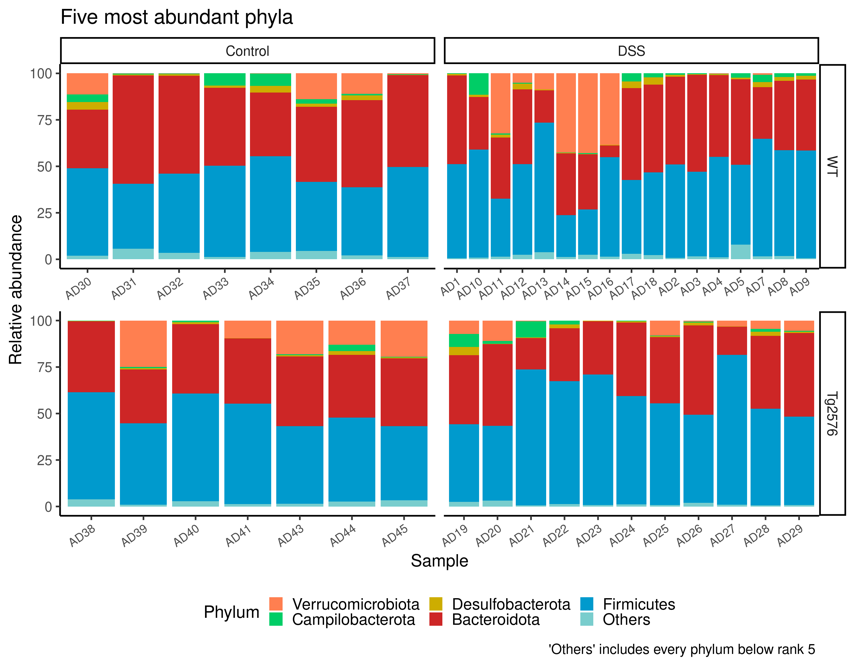


WT

Tg2576


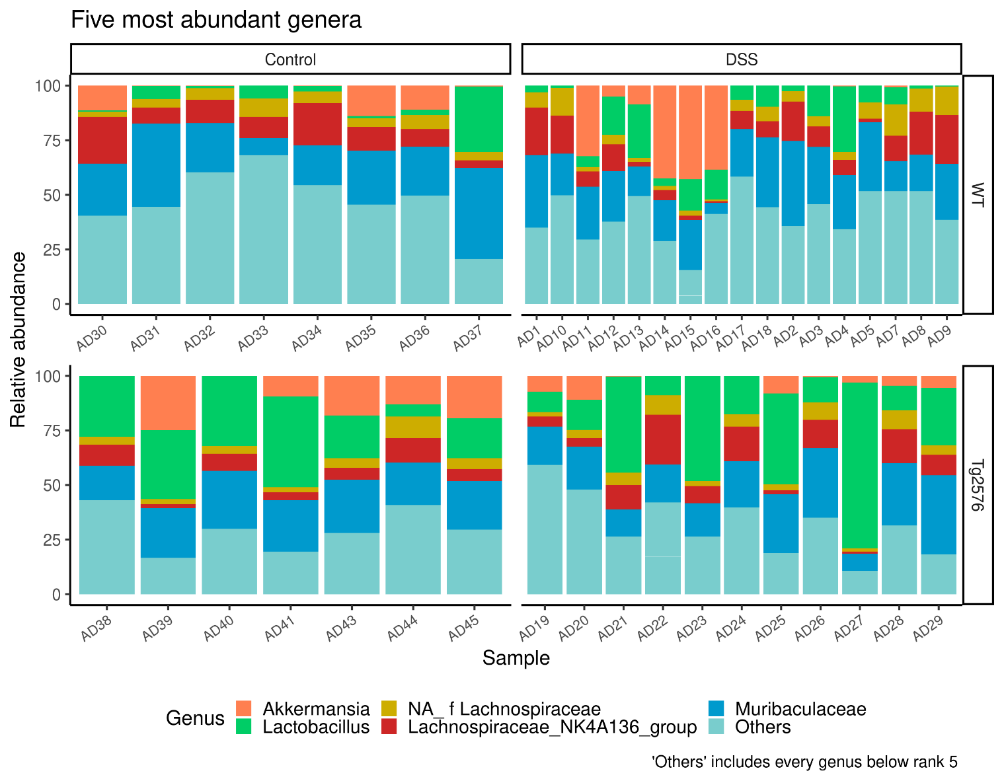


WT

Tg2576


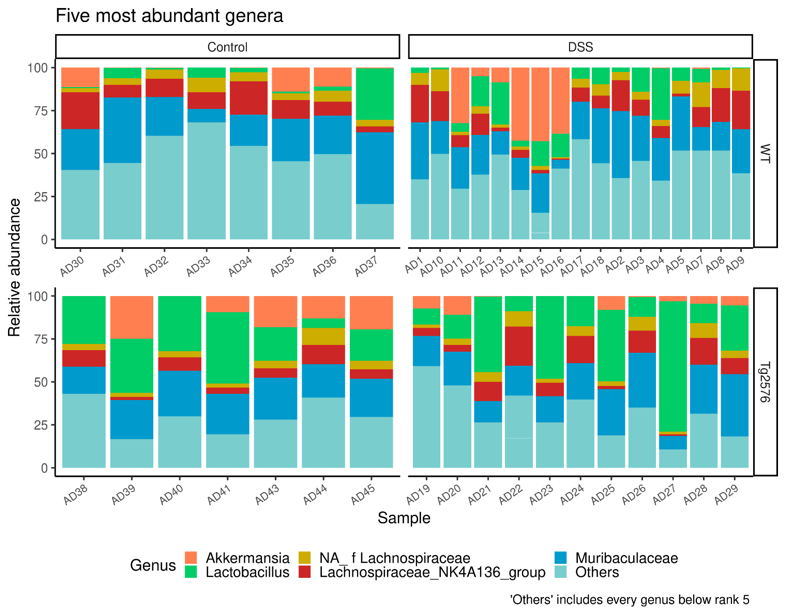

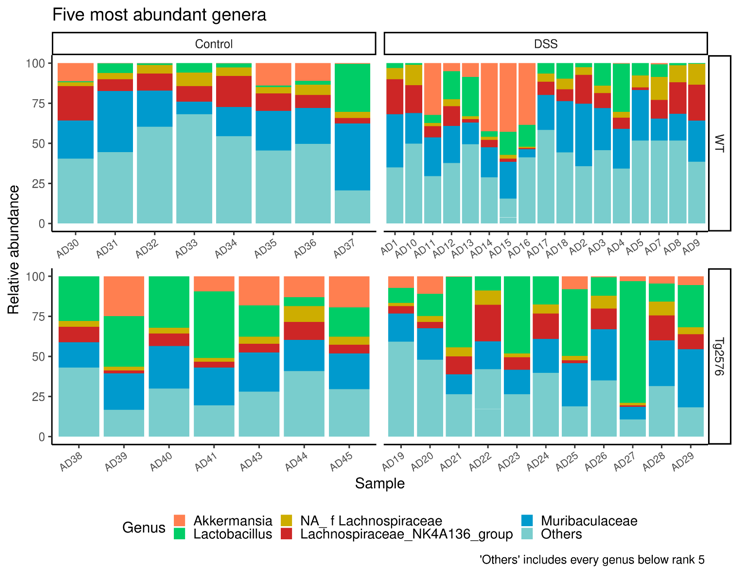


E


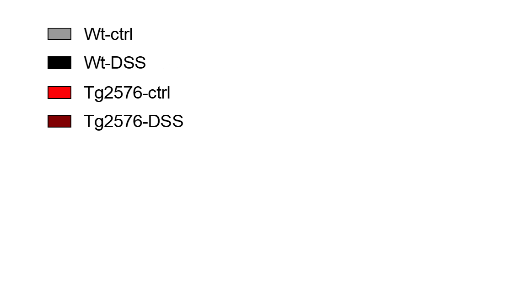

**Supplementary Figure 4.** Bar plots showing the percentage ot the total SCFA amount between groups. Analyses were assessed using the Kruskal-Wallis test.

| Total SCFAs | **WT-CONTROL** | **WT-DSS** | **p value** |
| --- | --- | --- | --- |
|  | 96.56 (1.44) | 97.56 (1.05) | 0.162 |
|  | **Wt-Ctrl** | **Tg2576-ctrl** | **p value** |
|  | 96.56 (1.44) | 92.08 (5.86) | 0.696 |
|  | **Wt-Ctrl** | **Tg2576-DSS** | **p value** |
|  | 96.56 (1.44) | 97.72 (1.50) | 0.188 |
|  | **Wt-DSS** | **Tg2576-ctrl** | **p value** |
|  | 97.56 (1.05) | 92,08 (5,86) | 0.063 |
|  | **Wt-DSS** | **Tg2576-DSS** | **p value** |
|  | 97.56 (1.05) | 97.72 (1.50) | 0.961 |
|  | **Tg2576-ctrl** | **Tg2576-DSS** | **p value** |
|  | 92,08 (5,86) | 97.72 (1.50) | 0.082 |

**Supplementary Table 3.** Total SCFA amount among Wt-ctrl and Tg2576 groups. Comparisons were assessed with the Mann-Whitney test and p-values less than 0.05 were considered statistically significant.


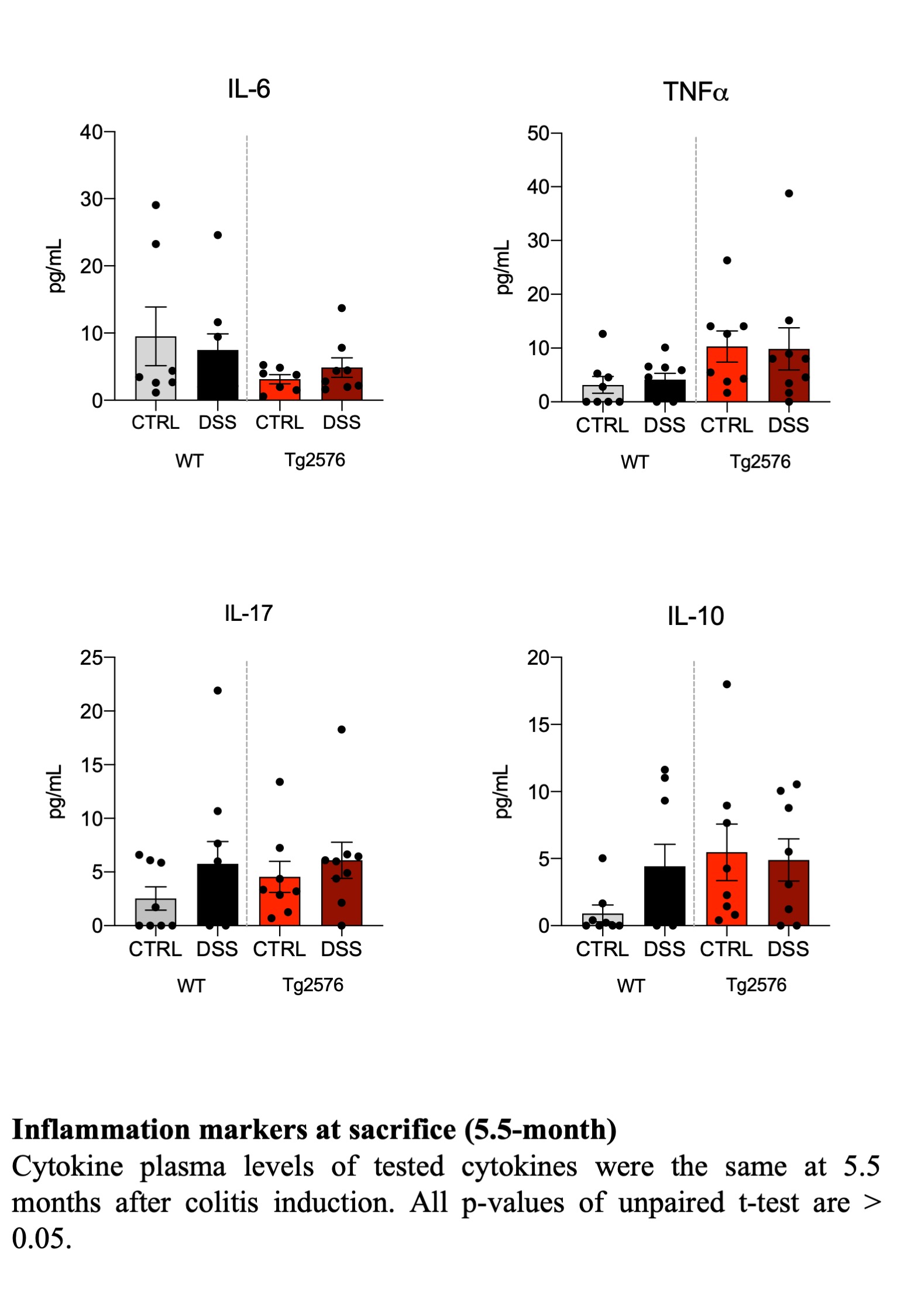


**Supplementary Figure 5. Inflammation markers at sacrifice (5.5-month).**

Cytokine plasma levels of tested cytokines were the same at 5.5 months after colitis induction. All p-values of unpaired t-test are > 0.05.
